# A recombineering pipeline to clone large and complex genes in Chlamydomonas

**DOI:** 10.1093/plcell/koab024

**Published:** 2021-02-02

**Authors:** Tom Z Emrich-Mills, Gary Yates, James Barrett, Philipp Girr, Irina Grouneva, Chun Sing Lau, Charlotte E Walker, Tsz Kam Kwok, John W Davey, Matthew P Johnson, Luke C M Mackinder

**Affiliations:** 1 Department of Biology, University of York, York YO10 5DD, UK; 2 Department Molecular Biology and Biotechnology, University of Sheffield, Sheffield S10 2TN, UK

## Abstract

The ability to clone genes has greatly advanced cell and molecular biology research, enabling researchers to generate fluorescent protein fusions for localization and confirm genetic causation by mutant complementation. Most gene cloning is polymerase chain reaction (PCR)�or DNA synthesis-dependent, which can become costly and technically challenging as genes increase in size, particularly if they contain complex regions. This has been a long-standing challenge for the *Chlamydomonas reinhardtii* research community, as this alga has a high percentage of genes containing complex sequence structures. Here we overcame these challenges by developing a recombineering pipeline for the rapid parallel cloning of genes from a Chlamydomonas bacterial artificial chromosome collection. To generate fluorescent protein fusions for localization, we applied the pipeline at both batch and high-throughput scales to 203 genes related to the Chlamydomonas CO_2_ concentrating mechanism (CCM), with an overall cloning success rate of 77%. Cloning success was independent of gene size and complexity, with cloned genes as large as 23 kb. Localization of a subset of CCM targets confirmed previous mass spectrometry data, identified new pyrenoid components, and enabled complementation of mutants. We provide vectors and detailed protocols to facilitate easy adoption of this technology, which we envision will open up new possibilities in algal and plant research.

## Teaser

A high-throughput system was developed to clone large, complex genes at high frequency and perform mutant complementation and protein tagging with a range of fluorophores in *Chlamydomonas reinhardtii*.

##  

**Figure:**
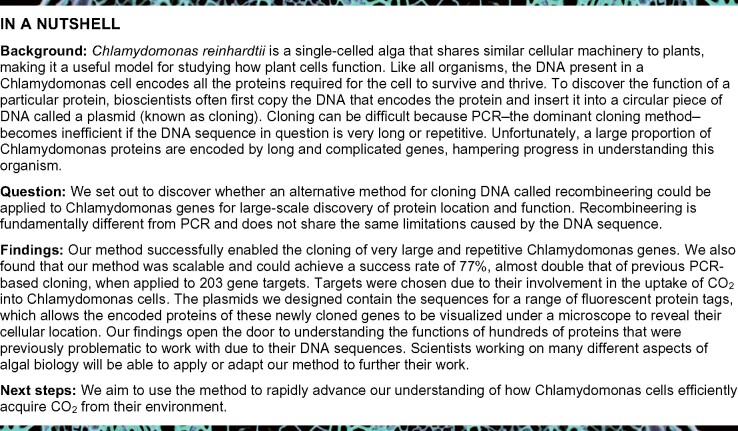


## Introduction

The unicellular alga *Chlamydomonas reinhardtii* (hereafter Chlamydomonas) is a widely used model organism for studying photosynthesis, biofuel production, human ciliopathies, flagella-powered motility, and cell cycle control (Salom� and Merchant, 2019). The nuclear, chloroplast, and mitochondrial genomes of Chlamydomonas have been sequenced, are well annotated and transformable. In addition, a variety of genetic resources are available to any institution, including a close-to-genome-saturating mutant library (Li et�al., 2019), extensive-omics-based data, and a wealth of molecular tools developed over decades by a dedicated research community (Salom� and Merchant, 2019). These collections, data, and tools are a vital resource for studies that aim to understand fundamental biological processes, to guide engineering efforts such as improved photosynthetic efficiency, and to enable efficient biomolecule production.

Reverse genetic approaches in Chlamydomonas often depend on localizing target proteins to understand spatial distribution and the complementation of mutants to link genotype to phenotype. Both of these methods generally rely on cloning a gene of interest into a plasmid from genomic DNA (gDNA) by PCR, followed by amplification in *Escherichia coli* and reintroduction into Chlamydomonas cells. PCR-based cloning from gDNA presents its own challenges and limitations that are particularly problematic when working with Chlamydomonas nuclear genes, which generally have a high guanine-cytosine (GC) content (68% in coding regions), contain one or more introns, and can include complex repeating regions (Merchant et�al., 2007). On the other hand, cloning from complementary DNA can result in low or no expression of target genes, most likely due to a lack of introns and regulatory elements (Lumbreras et�al., 1998; Schroda, 2019). Some of the challenges associated with PCR-based cloning can be circumvented via whole or partial gene synthesis followed by re-assembly using cloning strategies such as Golden Gate. Although the falling costs of gene synthesis make this a viable option for some genes, for many others the need to include introns, high GC content and high gene complexity, typical of the Chlamydomonas nuclear genome, results in synthesis failure or is prohibitively expensive. For example, the cloning of *SAGA1* (encoding StArch Granules Abnormal 1), a 16.7-kbp gene target, required over 12 months of work, included multiple gene synthesis failures, and ultimately had to be assembled from three synthesized fragments with 14 introns removed due to repetitive regions (Itakura et�al., 2019).

Improved Chlamydomonas target gene and foreign gene (collectively transgene) expression (e.g. *GFP*) have been achieved through strain optimization (Neupert et�al., 2009), the development of systems with linked transgene and antibiotic resistance gene expression (Rasala et�al., 2012; Onishi and Pringle, 2016), and an advanced understanding of transgene silencing (reviewed in Schroda, 2019). Furthermore, the release of the Chlamydomonas Golden Gate-based Modular Cloning kit has provided a cloning framework and a selection of genetic elements to enable laboratories to rapidly assemble and test transgene constructs (Crozet et�al., 2018). Independent of background strain and expression system, it is now clear that inserting or maintaining introns, correct codon usage, and promoter sequence are all critical for robust transgene expression (Barahimipour et al., 2015; L�pez-Paz et�al., 2017; Baier et�al., 2018; Weiner et�al., 2018; Schroda, 2019). These considerations have made the cloning of Chlamydomonas target genes directly from gDNA the community standard for mutant complementation and fluorescent protein tagging. However, there are considerable technical hurdles to overcome when working with the expression of large Chlamydomonas genes, predominantly caused by inefficient amplification of gDNA due to gene size, GC content, and the complexity of target genes (Sahdev et�al., 2007). Although modern polymerases have been engineered to overcome sequence challenges (Hommelsheim et�al., 2014), they may still suffer from replication slippage events, which are exacerbated by the presence of repetitive regions (Levinson and Gutman, 1987; Clarke et�al., 2001). In addition to considerations of size and complexity, cloning native genes based on current genome annotations can be complicated by the abundance of upstream transcription start sites corresponding to possible alternative open reading frames (ORFs; Cross, 2015) and, hence, potentially resulting in incorrect target gene cloning.

The results of a recent high-throughput localization study illustrate the challenges of PCR-based cloning of Chlamydomonas nuclear genes (Mackinder et�al., 2017). In Mackinder et�al. (2017), genes were PCR amplified from start site to stop site using gDNA as the template. Amplicons were then cloned in-frame via Gibson assembly with a fluorescent protein and a constitutive promoter and terminator, resulting in the successful cloning of 298 genes out of an attempted 624 (48% success rate), with most failures at the PCR amplification step. This relatively low success rate prompted us to develop a cloning platform based on recombination-mediated genetic engineering (recombineering) to allow Chlamydomonas genes to be cloned independently of size or sequence. Recombineering allows gene cloning to be performed by homologous recombination in *E. coli* without the need for PCR amplification of the template, making this technique predominantly independent of the size of the target region. Large-scale recombineering pipelines have been developed for bacterial artificial chromosome (BAC) and fosmid libraries from a broad range of organisms including the round worm *Caenorhabditis elegans* (Sarov et�al., 2006), fruit fly (*Drosophila melanogaster*; Sarov et�al., 2016), human and mice (Poser et�al., 2008), and *Arabidopsis thaliana* (Brumos et�al., 2020). Our pipeline involves making BAC-containing *E. coli* homologous recombination competent by introducing the recombinogenic viral proteins Red α, β, and γ from the bacteriophage lambda virus (Yu et�al., 2000; Copeland et�al., 2001) and then retrieving the target sequence by introducing 50-bp homology regions flanking a linearized plasmid.

We decided to apply our recombineering pipeline to an extended list of putative CO_2_ concentrating mechanism (CCM) genes. The CCM enhances photosynthesis by increasing the concentration of CO_2_ around Rubisco. To achieve this, Chlamydomonas actively accumulates inorganic carbon in the chloroplast and delivers it as CO_2_ to tightly packed Rubisco within the pyrenoid (Wang et�al., 2015). The pyrenoid is essential for CCM function in Chlamydomonas (Meyer et�al., 2012; Mackinder et�al., 2016). Due to the photosynthetic turbocharging properties of pyrenoid-based CCMs, there is growing interest in engineering them into crop plants to boost yields (Mackinder, 2017; Rae et�al., 2017). Recent studies have identified a large number of potential pyrenoid and CCM components (Mackinder et�al., 2017; Zhan et�al., 2018) that require functional characterization to understand their utility for future synthetic CCM engineering efforts. However, many of the genes encoding these components are challenging to clone due to size and sequence complexity, making localization and mutant complementation studies difficult.

Here, by applying our pipeline, we successfully cloned 157 CCM-related genes with their native promoters. Successful cloning using our system appears to be independent of target gene size, and many target genes have multiple complex features that would typically result in PCR failure. The average cloned region was 7.3 kbp, and target regions up to 22.7 kbp in size were successfully cloned. The inclusion of the native promoters ensures that any upstream ORFs (uORFs) have been incorporated. The localization of a subset of the proteins encoded by these genes allowed us to identify their diverse cellular locations, confirming previous interaction data (Mackinder et�al., 2017) and pyrenoid proteomic data (Mackinder et�al., 2016; Zhan et�al., 2018). We developed a series of recombineering vectors with a range of different fluorophores, epitope tags, and selection markers that can be used for protein localization, protein interaction, mutant complementation, and relative protein abundance studies. Our method takes 4 days to implement, is accessible for any laboratory equipped for molecular biology, and requires no specialized reagents or equipment. The BAC library used in this work and all developed vectors are available from the Chlamydomonas Resource Center. Finally, we provide a detailed protocol, allowing this method to be rapidly adopted by research laboratories to clone nuclear Chlamydomonas genes.

## Results

### Analysis of the Chlamydomonas genome highlights the challenges affecting PCR-based cloning

Cloning Chlamydomonas genes for successful localization and complementation studies often requires the amplification of complete ORFs from gDNA, spanning from their start site to their stop site including any introns (ATG-Stop). To gain a better understanding of the challenges involved in cloning Chlamydomonas genes, we performed a whole-genome analysis of gene size, complexity, intron prevalence, splice variants, and ATG-Stop primer suitability in Chlamydomonas, including comparisons to available datasets and other organisms.

#### Gene size

A major limitation of PCR-based cloning is the size of the target amplicon. Analysis of ATG-Stop cloning data from Mackinder et�al. (2017) for 624 genes using gDNA as a template and Phusion Hot Start II DNA polymerase (ThermoFisher Scientific, Waltham, MA, USA) revealed an association between cloning success and gene size; the average cloned ATG-Stop region was ∼2.3 kbp long, while the average uncloned region was ∼4.5 kbp (Mann–Whitney *U *=* *16,306, *P *<* *0.001, two-tailed). Extrapolation of PCR efficiency relative to target size from Mackinder et�al. (2017) to whole genes in the Chlamydomonas genome (version 5.5) indicated that 68% of genes would be technically challenging to clone via PCR-based methods (Figure�1A), predominantly due to a severe drop off in amplification efficiency for genes >3-kbp long. The largest amplified target in Mackinder et�al. (2017) was 8 kbp, and genes at least as large as 9.7 kbp have been cloned before (Kobayashi et�al., 2015), but this appears to be highly gene-specific. Alternative approaches exist to clone larger genes, such as testing a broad range of PCR conditions and DNA polymerases, amplification in fragments and re-stitching together, cloning from cDNA, and gene synthesis. While some of these approaches avoid the challenges presented here, they can be time consuming, costly, have low success rates, and may still result in no or poor expression.

**Figure 1:**
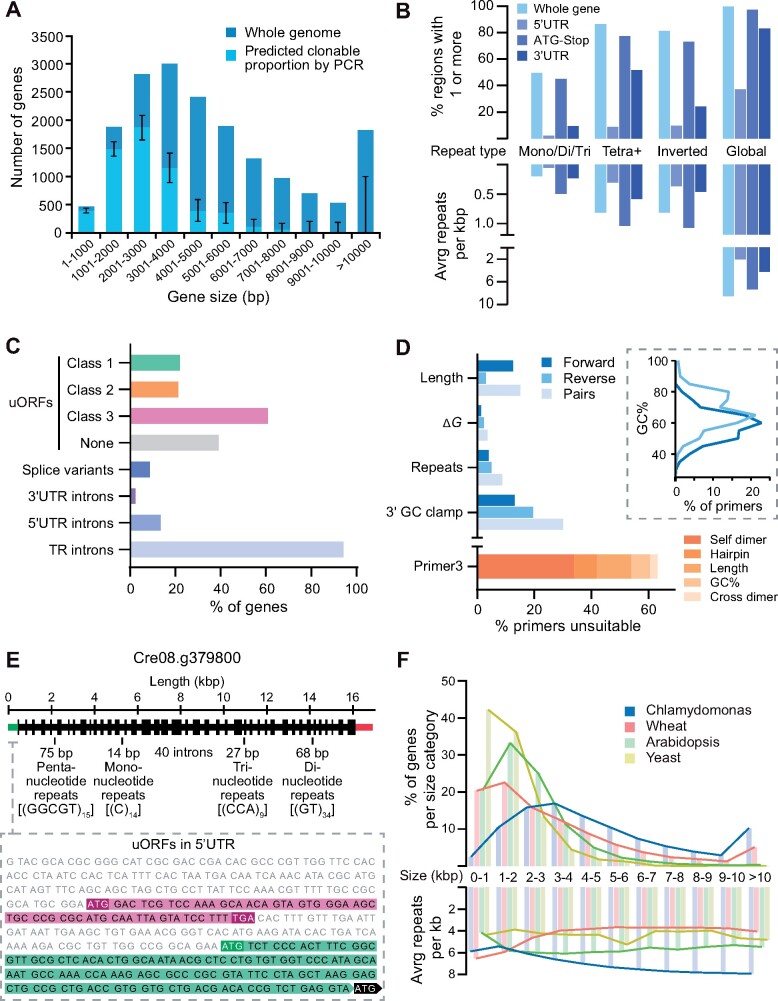
Chlamydomonas nuclear genes are often large, complex, or misannotated, affecting PCR-based cloning attempts and transgene expression success. A, The distribution of gene sizes for the 17,741 genes in the Chlamydomonas nuclear genome (dark blue). Gene sizes are measured from the start of the 5′-UTR to the end of the 3′-UTR. Within each size category, the predicted proportion amenable to PCR-based cloning is shown in light blue. These proportions were extrapolated from cloning success for 624 CCM-related genes from Mackinder et�al. (2017) in which PCR-based cloning was used to amplify the ATG-Stop region of each gene, excluding any UTRs. The strong size-dependence of ATG-Stop cloning efficiency seen in 2017 indicates that 68% of the genome would be challenging to clone. 95% confidence intervals for the predicted clonable proportions of each size category were calculated using the Wilson score interval method. No genes over 8,000 bp are predicted to be clonable by PCR although only a handful of regions of these sizes were tested in 2017 giving rise to the large confidence intervals for these categories. B, Genome-wide sequence complexity, as indicated by the presence of one or more repetitive sequences and frequency of repeats per kbp in each gene (pale blue). Values are also given for repeats localized to the 5′-UTR (light indigo), ATG-Stop (indigo), and 3′-UTR (dark indigo) within each gene. Note that while all 17,741 genes contain a start-to-stop region, not all genes contain a 5′-UTR and/or 3′-UTR, so the percentages presented for these are relative to totals of 17,721 and 17,717, respectively. Simple repeats are shown in the left three categories. Mono/di/tri refers to tandem repeats with a period length of one, two or three; tetra+ refers to all oligonucleotide tandem repeats with a period length of 4 or more and a total length ≥20 bp. Combining whole-gene counts for mono-, di-, tri-, and tetra+ produces an average value of 1.07 tandem repeats per kbp. Inverted repeats refer to short (20–210 bp) sequences that have the potential to form secondary structures by self-complementary base pairing. About 836 genes were free from detectable tandem and inverted repeats under our criteria, most of which are small, with an average length of 1,766 bp. Global repeats refer to repetitive sequences masked by the National Centre for Biotechnology Information (NCBI) WindowMasker program (Morgulis et�al., 2006), which includes both longer, non-adjacent sequences and shorter, simple repeats (see Methods section). All genes contained detectable repetitive regions using the default WindowMasker settings, with an average of 40.07 per gene. UTR data are based on gene models from Phytozome (version 5.5). C, Gene features that complicate correct transgene expression. Top four bars illustrate potential misannotation of functional start sites in the genome shown by the percentage of genes containing one or more uORFs of each class (see text). Note that some genes contain multiple classes of uORF. Shown below this is the percentage of Chlamydomonas genes with multiple transcript models (splice variants), and those containing introns in the UTRs and TRs (between start and stop codons). uORF data are from Cross (2015). Splice variant and intron data are based on gene models from Phytozome (version 5.5). D, Analysis of a set of ATG-Stop PCR primers designed to clone every gene in the genome from start to stop codon using gDNA as the template (Mackinder et�al., 2017). Many primers are predicted to be unsuitable for efficient PCR, as shown by the percentage of forward (dark blue) and reverse (light blue) primers that breach various recommended thresholds associated with good primer design. Pairs (pale blue) are shown for which one or both primers breach the respective thresholds. Thresholds shown pertain to length, secondary structure stability, tandem repeats, and 3′-GC content. The inset shows the distribution of GC content of primers in the dataset, illustrating a clear trend in higher GC content at the 3′-end of coding sequences. Below this, the given reason for rejection of primers by the Primer3 check_primers module is shown in orange. Dimer and hairpin values refer to primers rejected for “high end complementarity” and “high any complementarity” errors, respectively. E, Annotated gene structure of Cre08.g379800. The gene encodes a predicted protein of unknown function but shows examples of several sequence features that contribute to sequence complexity. The unspliced sequence is 16,892 bases long with a GC content of 64.3%. The 41 exons are shown as regions of increased thickness, with 40 introns between them, the annotated 5′-UTR in green (left) and the 3′-UTR in red (right). Labels denote selected examples of simple repeats throughout the gene. The inset shows the 5′-UTR sequence, displaying examples of two classes of uORFs (see text); Class 3 is highlighted in magenta and Class 1 in green. For simplicity, only one of the seven class 3 uORFs are shown in full. Cre08.g379800 was successfully cloned and tagged using recombineering. F, A comparison of gene size and complexity between Chlamydomonas, bread wheat (*Triticum aestivum*), *A. thaliana* and *Saccharomyces cerevisiae*. Gene sizes were binned as in (A), and the average number of global repeats kbp masked by the NCBI WindowMasker program was counted for genes in each size category (Morgulis et�al., 2006). Genes were measured from the start of the 5′-UTR to the end of the 3′-UTR.

#### Gene complexity

High GC content and the presence of numerous repetitive regions can make PCR-based cloning challenging. Analysis of data from Mackinder et�al. (2017) showed that the average GC content for successfully cloned targets by ATG-Stop PCR cloning was 61.4%, while the average GC content for unsuccessful targets was 64.3%—a value exceeded by over 41% of Chlamydomonas nuclear genes. To analyze the genome for repetitive regions, we determined the frequency of simple tandem repeats, inverted repeats, and larger, interspersed repeats between the start of the 5′-untranslated region (UTR) and the end of the 3′-UTR of each gene. Tandem repeats were assessed by counting individual regions that consist of consecutive mono-, di-, or trinucleotide repeats. Mononucleotide repeats shorter than 10 bp and regions of di- and trinucleotide repeats shorter than 20 bp were excluded. Some slight imperfections in the repeating pattern of a region were allowed, with regions that showed ≥90% identity (such as GGGGGTGGGG) included. Of the 17,741 coding genes in the nuclear genome, 8,810 contain one or more mono-, di-, or trinucleotide repeats (Figure�1B). In terms of prevalence per kilobase, the average Chlamydomonas gene contains 0.21 tandem repeats, whereas Arabidopsis contains 0.16 and the yeast *Saccharomyces cerevisiae* contains 0.10. Interestingly, if polynucleotide repeats with higher period numbers are counted as well (from tetranucleotide repeats to tandem repeating units of hundreds of base pairs), these values increase 5-fold for Chlamydomonas (1.07 per kbp), 2.5-fold for Arabidopsis (0.39 per kbp), and 3-fold for yeast (0.3 per kbp), highlighting the repetitive nature of the Chlamydomonas genome. We assessed inverted repeats by counting regions over 10-bp long that are followed closely downstream by their reverse complement, with some mismatches allowed so that regions with ≥90% identity were included. 14,454 genes contain one or more inverted repeats of this kind (Figure�1B), with an average of 0.93 repeats per kbp.

To further validate these findings, we analyzed nuclear gene sequences for repeats using WindowMasker, a program for detecting global repeats that include larger non-adjacent sequences as well as a diverse range of tandem repeats and inverted repeats (Morgulis et�al., 2006). With this expanded detection range, Chlamydomonas genes contain an average of 38.9 repeats (6.8 per kbp), whereas Arabidopsis contains 13.7 (5.5 per kbp) and yeast contains 6.0 (4.2 per kbp). On average, Chlamydomonas genes are more repetitive between their start and stop codons than in their untranslated regions (Figure�1B), although at least one repeat was detected by WindowMasker in 36.6% of 5′-UTRs and 87.6% of 3′-UTRs. Crucially, analysis of sequence data from Mackinder et�al. (2017) for 624 Chlamydomonas genes revealed an association between ATG-Stop PCR cloning success and repeat frequency: the average cloned ATG-Stop region contained 6.1 repeats per kbp, whereas the average uncloned region contained 7.5 repeats per kbp (Mann–Whitney *U *=* *24,110, *P *<* *0.001, two-tailed).

#### Mis-annotation of start sites

Another challenge associated with PCR-based and gene synthesis-based cloning is the presence of incorrectly annotated gene models, which leads to the cloning of a non-biologically relevant sequence. An analysis of transcript models in the Chlamydomonas genome showed that additional ATGs upstream of the annotated start site are highly prevalent (Cross, 2015; Figure�1C top 4 bars). Cross (2015) categorized these potential uORFs into three classes: Class 1 uORFs, which initiate in-frame with the annotated start site, potentially producing an N-terminal extension relative to the annotated gene model; Class 2 uORFs, which initiate out-of-frame with the annotated start site and terminate within the coding sequence; and Class 3 uORFs, which initiate and terminate within the 5′-UTR. Data from Cross (2015) on the presence of translation initiation sites (Kozak sequences) preceding class 1 uORFs suggest that approximately half are the correct translation initiation site *in vivo*. In a PCR-based approach where a constitutive promoter is used, cloning from the wrong ATG may result in an out-of-frame or truncated product, potentially removing essential signal sequences for correct targeting. Fifty-seven of the 298 successfully cloned genes from Mackinder et�al. (2017) contained a Class 1 in-frame ATG upstream of the cloned region; therefore, ∼10% of cloned regions may have encoded truncated protein products.

#### Introns, UTRs, and splice variants

Chlamydomonas genes have a relatively high frequency of introns, providing a further challenge for PCR-based cloning. The average gene contains 7.3 introns with an average intron length of 373 bp compared to an average exon length of 190 bp. Ninety-four percent of genes contain introns between their start and stop codons, 13% of genes contain one or more introns in their 5′-UTRs, and 3.4% have introns in their 3′-UTRs. ATG-Stop cloning would omit introns in UTRs, potentially missing critical regulatory information. Furthermore, ∼9% of genes are annotated with two or more transcript models that result from alternative splicing (Figure�1C). This variation would be missed by cloning from cDNA or by gene synthesis that excludes native introns.

#### Unsuitable primers

When performing ATG-Stop PCR cloning of either gDNA or cDNA, the flexibility of primer design is limited. Sequence analysis of a set of genome-wide primer pairs for ATG-Stop cloning (Mackinder et�al., 2017) indicates that primers are frequently of poor quality and unsuitable for efficient PCR. The average primer in the dataset had a predicted melting temperature (*T*_m_) of 69.2�C and an average GC content of 64.2%. The primer *T*_m_ and GC contents of Chlamydomonas genes are expected to be high compared to other organisms with less GC-rich genomes; however, many primers also breached the recommended thresholds pertaining to length, secondary structure formation, repetitive sequences, and 3′-GC content. Primers are shown in Figure�1D (blue bars) as having breached these four thresholds if (1) they were longer than 30 bp; (2) the free energy (Δ*G*) required to disrupt secondary structure formation (self-dimers, cross-dimers, or hairpins) was less than −9 kcal mol^−1^ at PCR-relevant annealing temperatures (66–72�C); (3) they contained mono- or dinucleotide repeats of 5 or more; or (4) their 3′-ends contained five or more consecutive G/C bases. A stricter set of thresholds is utilized by the Primer3 check_primers module (Rozen and Skaletsky, 2000), which resulted in the rejection of over 60% of individual primers in the dataset, even when the program was set to ignore predicted annealing temperatures (Figure�1D, orange bar). Under these settings, only 13% of pairs were free from detectable issues in both primers. Interestingly, there is a mismatch in GC content between forward and reverse primers, with reverse primers having a considerably higher GC content (Figure�1D, inset).

Many individual genes contain a range of the above features that result in challenges faced during PCR cloning or gene synthesis. Figure�1E shows a gene from chromosome 8 that exhibits several examples and was a target for recombineering. Cre08.g379800 is >16-kbp long with 40 introns and contains mono-, di-, tri-, and pentanucleotide repeat regions of ≥9 repeats. It also contains a potential misannotated upstream ATG in the 5′-UTR that could initiate a Class 1 uORF, as well as seven Class 3 uORFs (Cross, 2015). Structural information for Cre08.g379800 was obtained from the version 5.5 gene model currently available on Phytozome.

To investigate whether the challenges faced in Chlamydomonas were similar in other organisms, we analyzed gene size and gene complexity relative to gene size for the model eukaryote *S. cerevisiae*, the model plant Arabidopsis, and the ∼17 Gb hexaploid genome of bread wheat (*Triticum aestivum)*. As shown in Figure�1F, Chlamydomonas has a higher proportion of long genes and fewer short genes than the three other genomes tested, along with a considerably higher average gene size (5,322 bp versus 1,430 bp for yeast, 2,187 bp for Arabidopsis, and 3,521 bp for chromosome-assigned genes in wheat). Unlike wheat, Arabidopsis, and yeast, Chlamydomonas genes show a trend of increasing complexity per kilobase for longer genes (Figure�1F), which might be in line with the observation that average UTR length increases with increasing gene length (Salom� and Merchant, 2019).

### Development of our recombineering pipeline

To overcome the challenges associated with PCR-based cloning, we developed a high-throughput recombineering pipeline for large-scale parallel cloning of Chlamydomonas nuclear genes from BACs with their native promoter regions intact. During pipeline development, we decided to pursue a simplified 1-step DNA retrieval recombineering approach rather than a BAC editing approach (i.e. Poser et�al., 2008; Brumos et�al., 2020) for several reasons: (1) using a gene retrieval method allows all cloning to be performed in the BAC host *E. coli*, thereby avoiding the need for BAC purification, which can be timely and low yielding; (2) assembled constructs contain only the gene of interest, making them considerably smaller than the original BAC. This allows a medium copy origin of replication to be used, which improves the ease of handling, and the smaller constructs minimize DNA fragmentation during Chlamydomonas transformation (Zhang et�al., 2014); (3) BACs contain many genes, and therefore, additional copies of genes adjacent to the gene of interest could have an unwanted phenotypic impact on transformed Chlamydomonas lines; (4) the backbone of the available BAC collection lacks a suitable Chlamydomonas selection marker. Therefore, additional BAC editing to insert a suitable selection marker (Aksoy and Forest, 2019) or inefficient and poorly understood plasmid co-transformation strategies would be required for selection; and (5) a typical BAC engineering approach would require two recombination steps, which would increase the time needed for the pipeline, decrease pipeline efficiency, and add further challenges due to the repetitive nature of the Chlamydomonas genome.

The simplicity of our pipeline allows the entire process to be completed in 4 days using only generic reagents. The final recombineered construct is a vector containing the target region (typically including the native promoter, 5′-UTR, and ORF) recombined in-frame with a downstream fluorescent protein gene followed by the photosystem I subunit II (*PSAD*) terminator (see Figure�2 for a pipeline schematic and Supplemental Protocol 1 for details). Our pipeline has four key steps: (1) *E. coli* harboring a BAC containing the gene of interest is made recombination competent by transformation with the pRed vector containing the lambda viral *exo*, *beta*, and *gam* genes (*Redαβγ*) and *recA* (Sarov et�al., 2006; Figure�2A); (2) *Redαβγ* and *recA* are induced by arabinose treatment, followed by transformation with a linear tagging cassette including 50-bp homology arms to the target gene (Figure�2B); (3) kanamycin selection is performed to identify successful recombination events, and temperature-induced inhibition of the pRed pSC101 replication origin is performed to minimize further undesired recombination (Figure�2C); and (4) the plasmid is isolated and verified via restriction digest and junction sequencing (Figure�2D).

**Figure 2:**
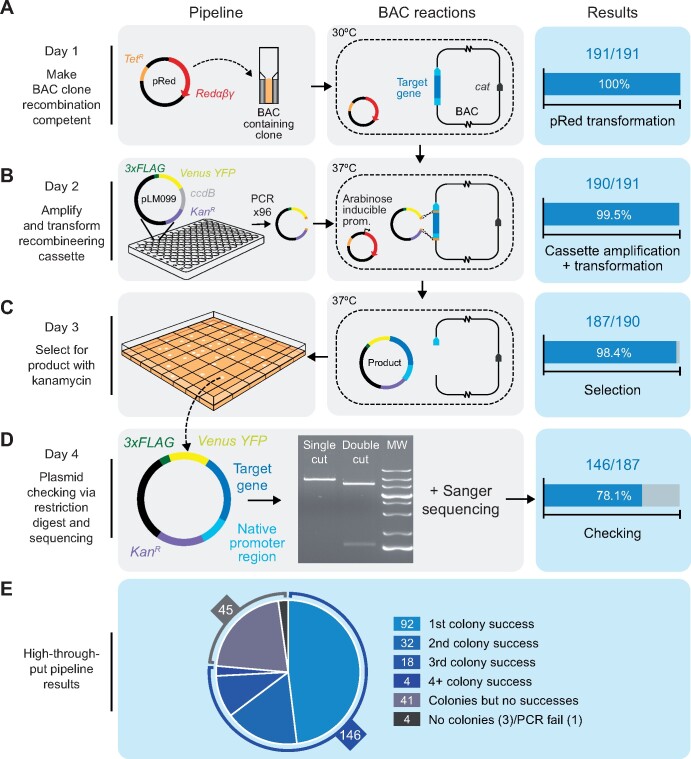
High-throughput recombineering pipeline for generating Venus-tagged fusion proteins with the native promoter regions intact. A, On Day 1, BAC clones containing target genes are made recombineering competent by transformation with the pRed plasmid, which encodes the viral recombinogenic *Redαβγ* genes and *recA* under the control of an arabinose inducible promoter. Transformation efficiency shown on the right-hand side relates to BAC clones that yielded colonies after selection with tetracycline and chloramphenicol. *Cat*: the chloramphenicol resistance gene in the backbone of every BAC clone in the BAC library. B, On or before Day 2, the recombineering cassette is amplified from pLM099 using primers that contain 50-bp homology arms complementary to regions flanking the target gene (shown in orange): one >2,000-bp upstream of the annotated ATG and one at the 3′-end of the coding sequence. On Day 2, BAC-containing cells are electrotransformed with the recombineering cassette after induction with l-arabinose. Recombination between the BAC and the cassette results in a plasmid product containing the target gene in frame with CrVenus-3xFLAG and under the control of its native promoter. Efficiency shown at this stage relates to PCRs that yielded efficient amplification of the recombineering cassette. C, On Day 3, colonies containing plasmid products are isolated. Efficiency at this stage relates to the number of transformations that yielded colonies after selection with kanamycin. D, On Day 4, plasmid products are extracted from cells, screened by enzymatic digestion and confirmed by sequencing. Efficiency shown at this stage relates to correct digest patterns with single and double cutting restriction enzymes. MW, molecular weight marker. E, Overall efficiency split into number of colonies screened via restriction digest. For 74% of target regions, the correct digest pattern was observed from plasmids isolated from the first, second or third colony picked per target. For 3% of targets, analyzing >3 colonies yielded the correct product.

The original tagging cassette consists of the codon-optimized yellow fluorescent protein�(YFP) gene *CrVenus*, a 3xFLAG tag, the *PSAD* terminator, the paromomycin selection marker (*AphVIII)*, the p15A medium-copy-number origin of replication, and the kanamycin resistance gene (*Kan^R^*). Amplification of the tagging cassette from pLM099 is performed using primers containing 50-bp homology arms corresponding to regions flanking the target gene; the forward primer is located at least 2,000-bp upstream of the start codon to encompass the native 5′-promoter and UTR and the reverse primer is located at the 3′-end of the coding region (immediately upstream of the stop codon). The annealing site of the reverse primer can easily be altered to amplify a cassette from pLM099 to clone genes without a fluorescent tag or with only the 3xFLAG tag (see Supplemental Protocol 1). To minimize false positives due to pLM099 carryover, pLM099 contains the control of cell death B (*ccdB*) counter-selection gene (Bernard and Couturier, 1992). In addition, the cassette includes an I-SceI restriction site. I-SceI has an 18-bp recognition site not found within the reference Chlamydomonas genome (strain CC-503) and allows the cassette to be linearized prior to transformation into Chlamydomonas.

We initially tested our pipeline on 12 targets. To ensure that the BAC library (available from the Chlamydomonas Resource Center; https://www.chlamycollection.org/) was correctly mapped, we performed PCR to check for the presence of the 5′- and 3′-ends of our target genes (Supplemental Figure 1A). We next implemented the pipeline using a small-scale batch protocol (Supplemental Protocol 1A). For all targets except one, plasmids isolated from most picked colonies gave a correct banding pattern after restriction digest (Supplemental Figure 1B). Sequence confirmation indicated that we successfully cloned 11 out of our 12 targets, resulting in a 92% success rate (Supplemental Figure 1C). To further expand the capabilities of our pipeline, we tested whether we could successfully recombineer a large and complex gene from a fosmid (available from the Chlamydomonas Resource Center). We targeted *SAGA1* (from fosmid VTP41289), which had previously been highly challenging to synthesize (see above; Itakura et�al., 2019) and was not available in the BAC library. Recombineered plasmids purified from three colonies all showed the correct restriction digestion pattern (Supplemental Figure 1D). Sequencing confirmed that the 19,601-bp target region, including 2,913-bp upstream of the predicted *SAGA1* start codon, was successfully cloned. Confident that our recombineering method was robust, we pursued the development of a large-scale pipeline that would allow the parallel tagging of genes with most steps achievable in 96-well format.

### Successful large-scale application of the recombineering pipeline

To test the efficiency of the pipeline, we shortlisted 191 genes that could be mapped to clones from the Chlamydomonas BAC library. To more easily identify BACs within the library that contain a target gene, we designed a Python script (BACSearcher; see Supplemental Protocol 2) and outputted the five smallest BACs for all targets in the genome (Supplemental Data Set 1), revealing that 86% of nuclear genes are covered by at least one BAC (87% if BACs are included that terminate within 3′-UTRs). BACSearcher can also be used for the automated design of primers containing 50′-bp homology regions to target genes in optimal positions; the script reports suitable 5′-homology regions 2,000- to 3,000-bp upstream of the annotated start codon and takes into account local DNA complexity features, including mono- and dinucleotide repeating runs and GC content. This feature can be easily modified to design 5′-homology regions further upstream of the target (see Supplemental Protocol 2A). The length of 50 bp is short enough to design into an oligonucleotide but long enough to be unlikely to share homology with more than one site within a BAC. Supplemental Data Set 1 includes sequences for the top five optimal 5′-homology regions for each target, all more than 2,000-bp upstream of the start codon, along with the corresponding 50 bp 3′-homology region. In addition, four pairs of primer sequences are included that can be used to check for the presence of each target in a BAC.

Our 191 targets were primarily chosen based on our 2017 association study for CCM components (Mackinder et�al., 2017), transcriptomics (Brueggeman et�al., 2012; Fang et�al., 2012), and pyrenoid proteomics (Mackinder et�al., 2016; Zhan et�al., 2018). Eighty-one genes previously targeted in 2017 were retried here by recombineering, this time with more than 2,000-bp upstream sequences included. Forty-one of these were previously unsuccessfully cloned by PCR and 40 were successfully cloned but were included here to compare the effect of retaining the native promoter. These included five targets that contain a Class 1 uORF (Cross, 2015) and so may have previously produced misleading localization data due to expression of a truncated protein. Selection of the remaining 110 targets was guided by new pyrenoid proteome (Zhan et�al., 2018) and CCM interactome data (Mackinder et�al., 2017). *Escherichia coli* strains containing the correct BAC, as identified by BACSearcher, were recovered from the BAC library and processed in parallel using 96-format culturing plates. To optimize the efficiency of our high-throughput pipeline, we successively ran the pipeline three times, removing successful targets once confirmed. A detailed protocol for the optimized high-throughput pipeline is provided in Supplemental Protocol 1B. In summary, 100% of our 191 target BAC lines were made recombination competent (Figure�2A) and out of the 191 target genes, one gene-specific tagging cassette failed to amplify (Figure�2B), likely due to the formation of secondary structure(s) within the 50-bp homology regions of the primers. Of the 190 that amplified successfully, 187 yielded colonies after selection with kanamycin (Figure�2C). Validation by enzymatic digestion confirmed that 146 of these lines contained correct recombineering plasmid products (Figure�2D). We extracted the recombineering plasmid products from the 146 successful lines and confirmed their junctions by Sanger sequencing. Our high-throughput pipeline had an overall efficiency of 76%, an average recombineered region length of 7,259 bp, and a maximum cloned length of 22,773 bp corresponding to gene Cre10.g427850 (Supplemental Data Set 2). Twenty-six target genes that were unsuccessfully cloned by PCR in 2017 were successfully cloned here by recombineering, and all five previously successful targets containing Class 1 uORFs retried here were successfully cloned.

During pipeline development, we found that optimizing bacterial growth prior to transformation with the recombineering cassette was critical (see notes in Supplemental Protocol 1). In addition, for 14 out of the 146 correctly recombineered lines in our high-throughput pipeline, the use of an alternative BAC from the library yielded success after an initial failure. For approximately half of the target genes, it was necessary to validate multiple colonies by enzymatic digest to rule out false positives; beginning with the 187 colony-producing lines from our high-throughput pipeline, picking just a single colony gave a 49% success rate, screening a second colony increased the success rate to 66%, and screening a third colony gave a 76% success rate. For a small proportion of targets, screening >3 colonies led to the identification of a correctly recombineered construct (Figure�2E). Restriction digest analysis of plasmids isolated from incorrectly assembled recombineering events suggested that cloning could fail for a broad range of reasons, including cassette recircularization, cassette duplication, cassette insertion into the BAC, or retrieval of incorrect target regions. Increasing homology arm length, using alternative homology arms, using alternative BACs, and using fosmids are potential solutions to overcome incorrect recombineering for specific targets. Supplemental Data Set 1 provides up to five options for homology arms and up to five available BACs per gene, and BACSearcher can be easily modified to increase homology arm length (see Supplemental Protocol 2A). Taken together with our 12 initial targets, we successfully cloned 157 out of 203 target regions from BACs using our recombineering pipeline, achieving an efficiency of 77%.

### Cloning success is size independent and tolerant of sequence complexity

To investigate if our recombineering approach was gene size and complexity independent, we compared our successful targets against unsuccessful targets (Figure�3). Here we defined a target region to mean the ATG-stop ORF for PCR-based cloning and the ATG-stop ORF plus an upstream region of >2,000 bp designed to encompass the 5′-UTR and native promoter for recombineering. There was no significant difference in the lengths of these regions between cloned and uncloned targets for recombineering (Figure�3A; Mann–Whitney *U *=* *3,303, *P *=* *0.38, two-tailed), indicating that our method is target size independent. This contrasts to the clear effect of target size on cloning success for our previous PCR-based cloning data (Figure�3A; Mackinder et�al., 2017). We then compared our cloning success to the number of simple and global repeats per kilobase in target regions. Our method appears to be far more tolerant of repetitive sequences than PCR-based cloning in terms of both the per-kilobase prevalence of simple and global repeats and the number of repeats per target region (Figure�3B and C). For our recombineering pipeline, there was no significant difference in the average number of repeats per kilobase between cloned and uncloned regions (Mann–Whitney *U = *3,129, *P *=* *0.17, two-tailed), whereas PCR-based cloning success was significantly reduced for targets with over ∼4.8 repeats per kbp (Figure�3B). For the most repetitive targets involved in our analysis (>9 repeats per kbp), our recombineering cloning efficiency remained above 60%, a rate over three times higher than that of PCR-based cloning (Figure�3B). Extrapolation of these data overlaid with the genome-wide distribution of repeat frequencies indicates that a large proportion of genes that are technically challenging for PCR-based cloning due to high repeat frequencies could be cloned by recombineering (Figure�3B).

**Figure 3:**
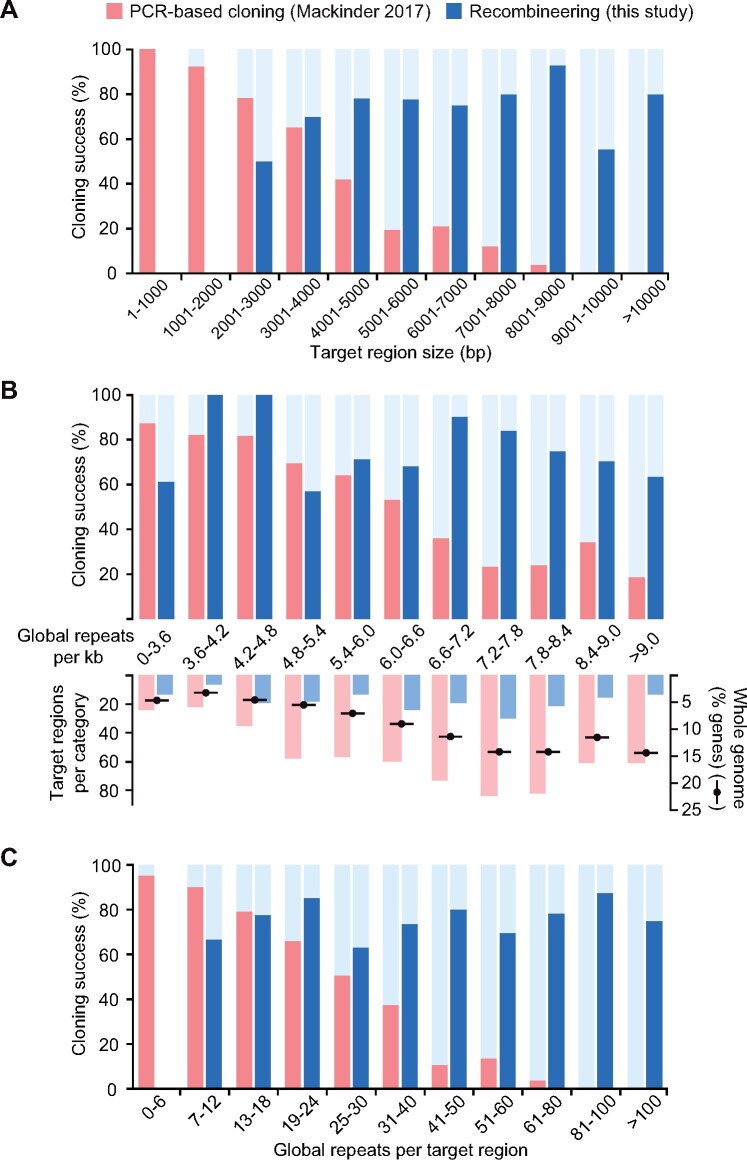
Our recombineering pipeline is target gene size independent and tolerant of sequence complexity. A, The size distribution of successfully PCR-cloned coding sequences (Mackinder et�al., 2017; red) or recombineered regions (this study; blue) are shown. Regions cloned by recombineering include ∼2 kbp of flanking DNA upstream of the annotated start codon to incorporate native 5′-promoter sequences. A severe drop in PCR-based cloning efficiency can be seen for templates >3-kbp long, whereas recombineering cloning efficiency does not show size dependency. No recombineering target regions were less than 2,000-bp long due to inclusion of native 5′-promoter sequences. B, As above but showing the dependence of cloning success on the per-kilobase frequency of repeats masked by the NCBI WindowMasker program with default settings (Morgulis et�al., 2006). The number of target regions per repeat category is shown beneath this, overlaid with the percentage of Chlamydomonas genes in each category. The distribution of targets for this study and our previous PCR-based cloning attempt (Mackinder et�al., 2017) gives a reasonably close representation of the whole-genome distribution. Almost a third of nuclear genes contain 7.2–8.4 repeats per kbp; this peak corresponds to a clear drop in PCR-based cloning efficiency, but to a high recombineering efficiency of 75–85%. Data for repeats per kbp were continuous and there are no values present in more than one category. C, As above but showing the number of simple and global repeats masked by WindowMasker per template. Data are binned to provide a higher resolution for the lower value categories, since the targets for PCR-based cloning were enriched in targets with low numbers of repeats. As in (A), a severe negative trend in PCR-based cloning efficiency can be seen, reflecting a strong positive correlation between repeat number and region size. No negative association is present for recombineering cloning efficiency, likely illustrating the benefit of avoiding size- and complexity-associated polymerase limitations. No recombineering target regions contained fewer than six repeats.

### Localization of Venus-tagged proteins

To assess the validity of the pipeline for localization studies, we transformed wild-type Chlamydomonas cells with a subset of linearized recombineering plasmid products tagged at the C-terminus with *CrVenus* (Figure�4A). Paromomycin-resistant colonies were directly screened for YFP fluorescence on transformation plates, picked, grown in Tris–phosphate (TP) minimal medium at air-levels of CO_2_ (∼0.04%), imaged by fluorescence microscopy to examine the localization pattern (Figure�4B;Supplemental Figure 2), and immunoblotted against the C-terminal 3xFLAG epitope to confirm fusion protein size (Supplemental Figure 2A). We selected the genes for transformation based on previous affinity purification mass spectrometry data (Mackinder et�al., 2017) and pyrenoid proteomics data (Mackinder et�al., 2016; Zhan et�al., 2018). The localization data support the proteomics data as we detected Photosystem I subunit F (PSAF), ISA1 (Isoamylase 1) and Chloroplast Stem-loop Binding Protein of 41 kDa B (CSP41B) in the pyrenoid. PSAF is a core transmembrane subunit of photosystem I. As expected, PSAF showed strong colocalization with chlorophyll outside of the pyrenoid; however, in addition, it clearly localized to the thylakoid tubules traversing the pyrenoid. Interestingly, in the pyrenoid tubules, the chlorophyll signal was minimal, particularly at the “pyrenoid tubule knot,” where the tubules converge (Engel et�al., 2015). These data, along with the finding that other PSI and PSII components localized to the pyrenoid tubules (Mackinder et�al., 2017), suggest that the tubules contain both PSI and PSII but that chlorophyll-containing light harvesting complexes found within the pyrenoid may be quenched or at low abundance. Tagged TAB2, a protein linked to early PSI assembly (Dauvill�e et�al., 2003) that was identified as an interactor with PSBP4 (a photosystem II subunit P-like protein) found within and at the periphery of the pyrenoid (Mackinder et�al., 2017), was also enriched at the pyrenoid. Interestingly, the location of TAB2 was not just restricted to the pyrenoid periphery, but it was also found within the pyrenoid, forming distinct small foci (Figure�4B). This finding suggests that early PSI assembly could be occurring within the pyrenoid as well as at the pyrenoid periphery (Uniacke and Zerges, 2009).

**Figure 4:**
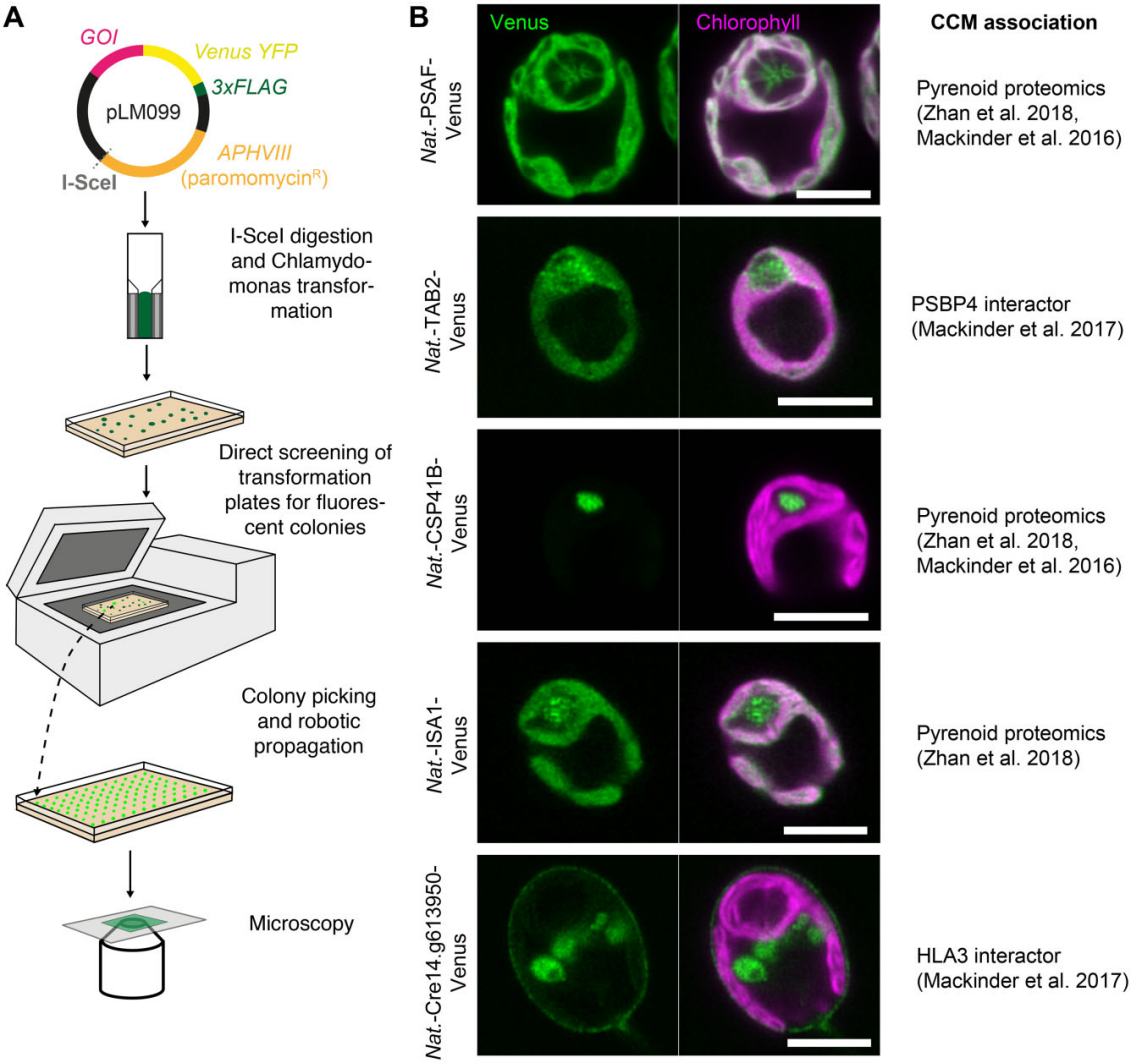
Transformation and localization of a subset of recombineered targets. A, Chlamydomonas transformation pipeline. The I-SceI cut site allows vector linearization prior to Chlamydomonas transformation via electroporation. Transformants are directly screened for fluorescence using a Typhoon scanner (GE Healthcare, San Diego, CA, USA) and then picked and propagated prior to imaging. GOI: gene of interest. B, The localizations of a subset of the recombineered target genes. Localizations agree with data from an affinity-purification followed by mass spectrometry study (Mackinder et�al., 2017) or pyrenoid proteomics (Zhan et�al., 2018 and/or Mackinder et�al., 2016). Scale bars: 5 �m.

CSP41B localized to the pyrenoid matrix, and analysis of the translated product of CSP41B showed that it belongs to a family of NAD-dependent epimerase/dehydratases (IPR001509) and contains a UDP-galactose 4-epimerase domain that may be involved in galactose metabolism. Its role in pyrenoid function is unclear. Localization of ISA1 showed that it was enriched in the pyrenoid, with an uneven distribution. ISA1 is a starch-debranching enzyme that is essential for starch synthesis, as *ISA1* deletion lines lack both chloroplast and pyrenoid starch (Mouille et�al., 1996). The presence of pyrenoid starch and its correct organization are critical for correct CCM function (Itakura et�al., 2019; Toyokawa et�al., 2020), as the absence of starch in an *ISA1* knockout mutant (4-D1) led to incorrect localization of the low-CO_2_-inducible (LCI) protein LCIB (see below), retarded growth at very low CO_2_ levels (0.01% v/v), and reduced inorganic carbon affinity (Toyokawa et�al., 2020). Interestingly, Toyokawa et�al. (2020) failed to obtain localization data for an ISA1-mCherry fusion driven by the *HSP70A/RBCS2* hybrid promoter.

Cre14.g613950 encodes a protein belonging to the ABC transporter family that was identified as an interactor of HLA3 (high light-activated gene 3; Mackinder et�al., 2017), a putative ${\text{HCO}}_{3}^{-}$ transporter located in the plasma membrane (Duanmu et�al., 2009; Gao et�al., 2015). Like HLA3, Cre14.g613950 shows a typical plasma membrane localization pattern, with YFP signal at the cell periphery and signal typical of the Golgi network. However, immunoblotting against the C-terminal 3xFLAG tag of Cre14.g613950 in two independent transformants showed a smaller molecular weight band than predicted (Supplemental Figure 2). This potentially indicates that the gene model for Cre14.g613950 is incorrect or that the protein undergoes post-translational cleavage, as seen for other CCM-related proteins that transit via the secretory pathway (Fukuzawa et�al., 1990; Tachiki et�al., 1992).

### Development of backbones with additional tags and markers

To further expand the functional application of our recombineering pipeline, we designed additional backbone vectors that enable protein tagging with the fluorophores mScarlet-i (Bindels et�al., 2017), mNeonGreen (Shaner et�al., 2013), and mTurquoise2 (Goedhart et�al., 2012) and that allow selection with hygromycin or zeocin (Figure�5, A and B). These vectors can be used to complement Chlamydomonas Library Project (CLiP) mutants that have been generated using the *AphVIII* marker conferring paromomycin resistance (Li et�al., 2016, 2019) and to express two or three differently tagged proteins within the same cell. For comparison, we tested these vectors on *LCI9* (Cre09.g394473), which encodes the low-CO_2_ inducible protein LCI9 that, via PCR-based cloning, we previously showed localizes to the pyrenoid periphery (Mackinder et�al., 2017). Recombineered *LCI9* was 7,160-bp long, including the native promoter region. All fluorophores displayed the same pyrenoid periphery localization pattern (Figure�5C), which agrees with the localization information obtained when LCI9 expression was driven by the *PSAD* promoter (Figure�5C bottom image; the *PSAD* promoter is here defined as the sequence spanning from 3 to 763-bp upstream of the *PSAD* start codon [Cre05.g238332], encompassing both the 5′-UTR and promoter region). These results further support the use of approximately 2,000-bp upstream regions as promoters for fusion protein expression.

**Figure 5:**
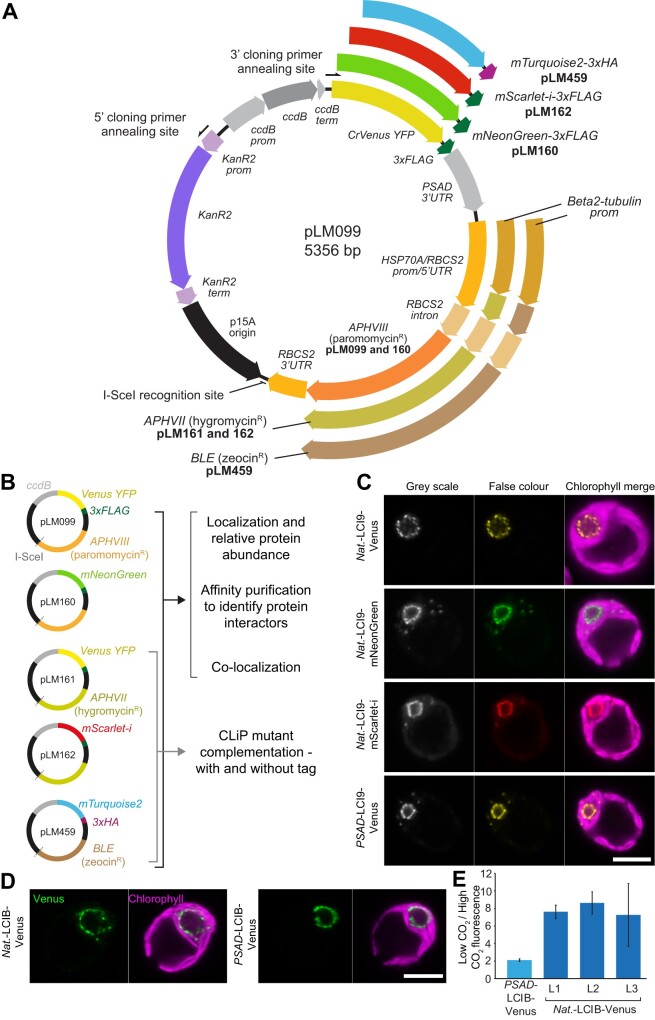
Development and application of different recombineering vectors to obtain novel biological insights into Chlamydomonas biology. A, Plasmid map for pLM099 and derivative recombineering vectors. PCR amplification with 5′- and 3′-cloning primers at the annealing sites shown results in a ∼4.6-kbp linear cassette for recombineering target genes in-frame with a fluorescent protein and affinity tag. For each recombineering vector, the fluorescent protein sequence is preceded by a flexible linker (GGLGGSGGR) and followed by a tri-glycine linker prior to the affinity tag. The *PSAD* 3′-UTR terminates all four fluorescent protein-affinity tag cassettes. The *RBCS2* 3′-UTR terminates all three Chlamydomonas selection cassettes. The same *RBCS2* intron is present in all three Chlamydomonas selection cassettes but is only inter-exonic in the hygromycin and zeocin-resistance cassettes. B Additional vectors for tagging with different fluorophores and for complementation of Chlamydomonas library mutants generated using insertion of the *AphVIII* paromomycin resistant gene. C, Localization of LCI9 with different fluorescence protein tags. *LCI9* was recombineered with its native promoter (*Nat.*) using pLM099, pLM160, and pLM162. A previously developed line cloned by PCR and using the constitutively expressed *PSAD* promoter is shown for comparison (*PSAD*-LCI9-Venus). Scale bar: 5 �m. D, A comparison of the low CO_2_ upregulated gene *LCIB* cloned with its native promoter via recombineering versus *LCIB* under the control of the constitutive *PSAD* promoter. Cells were grown and imaged at atmospheric CO_2_ levels. Scale bar: 5 �m. E, Relative change in LCIB-Venus fluorescence between high (3% v/v) and low (0.04% v/v) CO_2_ when expressed from the constitutive *PSAD* promoter versus expression from the native *LCIB* promoter. Data are shown for three independent native *LCIB* promoter lines (L1–L3). Error bars are standard error of the mean.

To further confirm that the localizations of proteins driven by their native promoters do not differ from those driven by the constitutive *PSAD* promoter, we compared the localizations of *native*-LCIB-Venus and *PSAD*-LCIB-Venus. LCIB is an essential CCM component that shows dynamic relocalization to the pyrenoid periphery at CO_2_ levels <0.04% (Yamano et�al., 2010). LCIB expressed from its endogenous promoter localized to the pyrenoid periphery under ambient CO_2_ levels (~0.04% v/v), in full agreement with the localization data when LCIB expression was driven by the constitutive *PSAD* promoter (Figure�5D).

Finally, we tested whether our recombineering pipeline could be used to successfully complement a CLiP mutant. We transformed *native-LCIB-Venus* (cloned into pLM161, containing the *APHVII* gene conferring hygromycin resistance) into a CLiP *lcib* mutant (LMJ.RY0402.215132) and examined the phenotypes of four transformants showing Venus fluorescence. All four transformants showed a typical pyrenoid peripheral localization pattern when grown at very low CO_2_ levels, and all rescued the *lcib* mutant phenotype to varying degrees, with *lcib:LCIB-Venus-1* showing complete rescue (Supplemental Figure 3).

### Maintaining the native promoter enables relative protein abundances to be monitored

As our pipeline retains the native promoter of the target gene, we hypothesized that fluorescence output would be representative of relative changes in protein abundance in response to environmental conditions. To test this, we grew lines with *LCIB* driven by either the constitutive *PSAD* promoter (*PSAD-*LCIB-Venus) or its native promoter (*Native*-LCIB-Venus). LCIB-Venus signal stayed relatively constant between high (3% v/v) and low (0.04% v/v) CO_2_ conditions when LCIB was expressed from the *PSAD* promoter (*PSAD-*LCIB-Venus), but showed an approximately eight-fold increase between these conditions when the native promoter was used, with this change consistent across three independently transformed lines (Figure�5E). This finding agrees with previous immunoblotting data, in which a comparable fold increase was seen in LCIB abundance when cells were transferred from high CO_2_ to low CO_2_ conditions (Yamano et�al., 2010). This indicates that our recombineering lines can be used to monitor relative protein abundance across different growth conditions.

## DISCUSSION

We have established a rapid recombineering-based method to clone large and complex Chlamydomonas genes from BACs. Our approach circumvents the challenges associated with cloning large, GC-rich, and complex genes that are prevalent in Chlamydomonas. We demonstrated that the method could be applied for small batch cloning as well as 96-well high-throughput cloning. Our overall cloning success rate (combined batch and high-throughput results) was 77%, a value considerably higher than that of our previous PCR-based high-throughput cloning pipeline (48%), which was inflated due to an enrichment of small target genes. Our overall success rate is slightly lower compared to recombineering pipelines in other organisms, with success rates of 89% achieved in *C. elegans* (Sarov et�al., 2012) and ∼93% in Arabidopsis (Brumos et�al., 2020). This reduced overall efficiency is likely due to the complexity of the Chlamydomonas genome (Figure�1), as DNA secondary structure was previously linked to recombineering failure (Nelms and Labosky, 2011). We expect a higher success rate when the pipeline is applied to a smaller number of samples, since it is easier to optimize bacterial growth prior to electrotransformation on a per-sample basis if there are fewer samples to manage. This may be evidenced by our successful cloning of 11 out of 12 targets in an initial batch-scale pipeline attempt (Supplemental Figure 1), although the sample size is insufficient to generalize with confidence.

To enable expression of multiple fluorophores simultaneously and for the complementation of CLiP mutants, we designed a series of vectors with modern fluorophores and varying selection markers and demonstrated their performance in Chlamydomonas (Figure�5). The presence of either 3xFLAG or 3xHA tag allows these vectors to be used for affinity purification to explore interacting partners of tagged proteins. Different fluorophore pairs (i.e. mNeonGreen and mScarlet-i) could also be used for FRET-based studies to explore protein–protein interactions. In addition, all vectors can be used to clone genes without fluorescence tags or with only short affinity tags (3xFLAG and 3xHA).

Due to the size independence of our method, we could maintain the native promoters of target genes. For two genes, *LCI9* and *LCIB*, there were no noticeable differences in protein localization between native promoter-driven expression and *PSAD* promoter-driven expression. Interestingly, using a native promoter allows relative protein abundance to be tracked between conditions (Figure�5E). Once validated, acquiring relative abundance data is straightforward and can be easily parallelized. This allows relative protein abundance to be tracked in real-time across a broad range of conditions. Future experiments could include tracking relative protein abundance in 96-well libraries of tagged proteins in response to a perturbation (i.e. high to low CO_2_ transition). This would be highly supportive of available transcriptomic and proteomic data sets and would provide novel insights into cellular processes (Mettler et�al., 2014; Zones et�al., 2015; Strenkert et�al., 2019). Although our relative abundance data for LCIB appear to closely reflect the immunoblotting data, it should be noted that using a native promoter may not always fully reflect native changes. This discrepancy could be due to insertional effects caused by integration into transcriptionally unfavorable regions of the genome and the absence of *cis*-regulatory regions in the recombineered construct or by transcriptional silencing (Schroda, 2019). At the protein level, fluorescent protein folding time could affect protein stability and turnover, and the presence of the fused fluorescence protein could affect function or multi-subunit assembly.

While our approach allows the native promoter, 5′-UTR, and ORF to be cloned, the native 3′-UTR is not maintained. This could be addressed through a two-step recombineering pipeline where the tag is first inserted into the BAC at the desired location and the markers are then removed via a Flp-*FRT* recombinase system (Sarov et�al., 2006; Brumos et�al., 2020). The edited target gene could then be retrieved into a final Chlamydomonas expression vector. When establishing our pipeline, we decided not to pursue this strategy to maximize the success rate by limiting the number of steps, with a focus on developing a simple, easy to apply approach. In addition, while we have focused on C-terminal tagging, as this allows N-terminal transit peptides required for organelle targeting to be conserved, our recombineering pipeline could be applied for N-terminal tagging by modifying our cloning vectors with a constitutive promoter and N-terminal tag.

The simplicity of our framework and vector design could be adopted for other organisms with relative ease provided a BAC or fosmid library and efficient transformation protocols are available. Multiple features of our recombineering cassette could make adaptation to different organisms relatively straightforward, such as the use of ccdB counter-selection and the rare I-SceI recognition site used for linearization of the recombineering cassette prior to transformation. For organisms in which selection with paromomycin, hygromycin, or zeocin is ineffective or the *AphVII*, *AphVIII*, or *BLE* genes included in the pLM099-derived cassettes cannot be utilized, alternative selection genes can be quickly incorporated by restriction-ligation using flanks containing KpnI and I-SceI recognition sites at the 5′- and 3′-ends, respectively.

One limitation we encountered was that only 86% of nuclear genes are covered by the BAC library. However, this value only takes into account ∼73% of BACs, with the remaining BACs potentially incorrectly mapped to the current version of the Chlamydomonas genome (see Supplemental Protocol 2B). Our analysis suggests that the true percentage of genes covered could be higher than 86%, but confirming this may require a careful re-mapping of the BAC library. A promising solution is cloning from fosmids. We demonstrated that our pipeline can be successfully used for cloning from fosmids, and a Chlamydomonas fosmid library is now available (released July 2020; Chlamydomonas Resource Center). The use of fosmids, with smaller DNA fragments compared to BACs, could help improve the efficiency of our pipeline by reducing off-target recombination between the PCR-amplified cassette and the BAC or by reducing recombination between two repetitive regions of the BAC. In addition, the fosmid library is expected to have close to 100% genome coverage.

Our recombineering approach allowed us to efficiently clone large and complex genes, which could not be achieved via PCR-based cloning. This method opens the door to a better understanding of the functional roles of a large fraction of the Chlamydomonas genome through protein localization, protein–protein interaction studies, real-time monitoring of relative protein abundance, and complementation of mutants (e.g. random insertion and CRISPR/Cas-generated mutants). In addition, it provides a highly complementary method to the recently released CLiP mutant collection.

## Materials and methods

### Plasmid and cassette construction

Fragments for pLM099 were amplified by PCR (Phusion Hotstart II polymerase, ThermoFisher Scientific, Waltham, MA, USA) from the following plasmids: Venus-3xFLAG, PSAD terminator and *AphVIII* from pLM005 (Mackinder et�al., 2017); the p15A origin of replication from pNPC2; the *Kan^R^* resistance gene from pLM007; the counter-selection *ccdB* gene from Gateway pDONR221 Vector (ThermoFisher Scientific). The resulting amplicons were gel purified (MinElute Gel Extraction Kit, Qiagen, Valencia, CA, USA) and assembled by Gibson assembly (see Figure�5A for detailed map). pLM160 was constructed from pLM099 to replace CrVenus with mNeonGreen (Shaner et�al., 2013), and pLM161 was constructed from pLM099 to replace the paromomycin resistance gene (*AphVIII)* with the hygromycin resistance gene (*AphVII)*. pLM162 was constructed from pLM161 with the synthetic fluorophore mScarlet-i (Bindels et�al., 2017) replacing CrVenus. pLM459 was constructed from pLM161 to replace CrVenus with mTurquoise2 (Goedhart et�al., 2012), the 3xFLAG with the 3xHA haemagglutinin tag, and *AphVII* with the zeocin resistance gene (*Sh ble)*. Gene-specific cloning primers were designed to amplify a ∼4.6 kbp cassette from the recombineering vectors pLM099, 160, 161, 162, and 459 (Figure�5), excluding *ccdB*, and providing 50 bp of sequence homology to the target gene an average of ∼2,500-bp upstream of the 5′-UTR and directly upstream of the stop codon. This enables the retrieval of each target gene into the cassette in frame with a fluorescent tag and with the native promoter region intact. All oligonucleotide and plasmid sequences can be found in Supplemental Data Sets 3 and 4.

### Culturing

*Escherichia coli* cells were cultured in lysogeny broth (LB) or yeast extract nutrient broth (YENB) at 37�C unless they contained the temperature-sensitive pSC101-BAD-gbaA-tet (pRed), in which case 30�C was used. All DNA for transformation was introduced by electroporation and transformants were recovered in super optimal broth with catabolite repression (SOC). DH10B cells containing fragments of the Chlamydomonas genome in the form of BACs were obtained from the Clemson University Genomics Institute (now distributed by the Chlamydomonas Resource Center, University of Minnesota, Minnesota, MN, USA). DB3.1 cells expressing the *ccdB* antidote gene *ccdA* were obtained from ThermoFisher Scientific and used for maintenance of the recombineering vectors.

Chlamydomonas wild-type cells (strain CC-4533) were cultured in Tris–acetate–phosphate (TAP) medium with revised Hutner’s trace elements (Kropat et�al., 2011) and illuminated by white fluorescent light. Assembled recombineering vectors were prepared for transformation into Chlamydomonas by restriction digest with I-SceI endonuclease (NEB). Transformation and selection of fluorescence lines were performed in accordance with Mackinder et�al. (2017) using a Typhoon Trio fluorescence scanner (GE Healthcare, Madison, WI, USA). Viable Chlamydomonas transformants were screened for CrVenus and mNeonGreen fluorescence emission at 555/20 nm, and for mScarlet-i at 615/12 nm. Several strains emitting the strongest fluorescence for each line were picked. The average number of fluorescent colonies for recombineered Venus fusion proteins with their native promoter was ∼10%; however, this varied considerably between constructs (PSAF (10/134) 7%, TAB2 (6/44) 13.6%, CSP41B (6/43) 13.9%, ISA1 (25/297) 8%, Cre14.g613950 (2/22) 9%, LCI9 (6/25) 24%, and LCIB (6/19) 31.5%). Picked fluorescent strains were cultured in Tris–phosphate (TP) minimal medium under ambient CO_2_ (∼0.04%) conditions and imaged by fluorescent microscopy to visualize protein localization. To ensure that the determined localizations were not due to in-frame integration of a fluorophore-containing fragment of the cassette with another gene, we confirmed localization in at least two independent transformants and performed immunoblotting against the 3xFLAG epitope to confirm the expected fusion protein size.

For spot tests, cells were grown to ∼8 � 10^6^ cells mL^−1^ in TAP at ∼50 �mol photons m^–2^ s^–1^, washed with TP, and serial diluted in TP prior to spotting 1,000, 100, and 10 cells on TP 1.5% agar plates. Replica plates were incubated in 0.04% or 3% CO_2_ chambers for 24 h at 50 �mol photons m^–2^s^–1^, then 24 h at 150 �mol photons m^–2^ s^–1^, followed by 48 h at 300 �mol photons m^–2^ s^–1^ prior to imaging.

### Protein extraction and immunoblotting

Lines expressing recombineered fusion proteins were cultured in 50 mL TAP medium containing 5 �g mL^-1^ paromomycin to a cell density of ∼2 � 10^6^ cells mL^–1^. Cells were harvested by centrifugation at 5,000*g* for 10 min at room temperature. The supernatant was discarded, and the pellet was resuspended in 500 �L of protein extraction buffer (20 mM Tris–HCl pH 7.5, 5 mM MgCl_2_, 300 mM NaCl, 5 mM Dithiothreitol�[DTT], 0.1% Triton-X100, Roche protease inhibitor) and flash frozen in liquid nitrogen in 100 �L aliquots. Cells were thawed on ice and flash frozen again before a final thaw on ice. Samples were then centrifuged at 17,000*g* for 15 min at 4�C to separate the soluble and insoluble fractions. The soluble supernatant was transferred to a new tube, mixed 1:1 with 2� Laemmli buffer containing β-mercaptoethanol, and heated at 80�C for 10 min prior to sodium dodecyl sulfate–polyacrylamide gel electrophoresis (SDS–PAGE).

About 15–30 �L of each sample was loaded onto a 10% mini-protean TGX gel (Bio-Rad) and transferred to a polyvinylidene difluoride membrane via semi-dry transfer (10V, 60 min). Fusion proteins were immuno-detected using the monoclonal anti-flag M2 antibody (1:1,000; Sigma-Aldrich, St Louis, MO, USA; catalog # F1804), followed by Alexa-Fluor 555 goat anti-mouse secondary antibody (1:10,000; Invitrogen, Carlsbad, CA, USA; catalog # A-21422). The membrane was imaged using a Typhoon 5 Scanner.

### Microscopy

Sample preparation for microscopy was performed as per (Mackinder et�al., 2017). Images were acquired using a Zeiss LSM880 confocal microscope on an Axio Observer Z1 invert, equipped with a 63� 1.40 NA oil planapochromat lens. Images were analyzed using ZEN 2.1 software (Zeiss, San Diego, CA, USA) and FIJI. Excitation and emission filter settings were as follows: Venus and mNeonGreen, 514 nm excitation, 525–550 nm emission; mScarlet-i, 561 nm excitation, 580–600 nm emission; and chlorophyll, 561 nm excitation, 665–705 nm emission.

### Plate reader assay

To monitor changes in fluorescence in response to CO_2_, three independent *native-*LCIB-Venus lines, a single *PSAD-*LCIB-Venus line, and wild-type (WT) were grown in TP bubbled at low CO_2_ (0.04% v/v) or high CO_2_ (3% v/v) conditions at 300 �mol photons m^–2^ s^–1^. Four samples per line were aliquoted into a 96-well plate and chlorophyll (excitation 625/34, emission 692/50) and Venus (excitation 504/10, emission 540/12) fluorescence was immediately measured using a BMG Labtech Clariostar Plate Reader. Venus fluorescence was normalized by chlorophyll, and WT background was then subtracted. The average fluorescence value under low CO_2_ conditions was divided by the average fluorescence value under high CO_2_ conditions for each line. Error was calculated by the propagation of variance across both low and high CO_2_ values and is shown as the standard error of the mean.

### Recombineering procedure for one-step subcloning and tagging

The following outlines the batch-scale recombineering protocol. Extended batch and multi-well plate-scale recombineering protocols are provided in Supplemental Protocol 1.

For each target, a recombineering cassette was amplified from plasmid pLM099 (Phusion Hotstart II polymerase, ThermoFisher Scientific, Waltham, MA, USA) using primers containing 50-bp homology arms, one homologous to a region upstream of the annotated start codon of the target gene, and one homologous to the 3′-end of the coding sequence (excluding the stop codon). The resulting PCR product was gel purified (MinElute Gel Extraction Kit, Qiagen, Valencia, CA, USA) and its concentration measured using a NanoDrop spectrophotometer. Upstream region lengths ranged from 1,000 to 4,000 bp from the start codon, with an average of ∼2,500 bp. For two genes, Cre04.g220200 and Cre16.g678661, the first 50 bp of the 5′-UTR was used as the upstream homology region due to BAC coverage limitations.

The pRed plasmid pSC101-BAD-gbaA-tet was extracted from *E. coli* cells grown overnight at 30�C (Plasmid Mini Kit, Qiagen, Valencia, CA, USA) and its concentration measured by NanoDrop. *Escherichia coli* cells harboring a BAC containing the target gene were recovered from the Chlamydomonas BAC library and used to inoculate 20 mL of YENB medium containing 12.5 μg mL^−1^ chloramphenicol, followed by overnight growth in a 50 mL conical flask at 37�C with vigorous shaking. After 16 h of growth, 120 μL of the culture was used to inoculate 4 mL of fresh YENB containing 12.5 μg mL^-1^ chloramphenicol. This was grown for ∼2 h at 37�C until an optical density (OD_600_) of 2 was reached. About 2 mL of the culture was then incubated on ice for 2 min, followed by centrifugation at 5,000*g* for 10 min at 4�C. After removing the supernatant, the pellet was placed back on ice and washed by resuspension in 1 mL of chilled 10% glycerol, followed immediately by centrifugation at 5,000*g* for 10 min at 4�C. The resulting supernatant was removed, and the pellet was placed back on ice and resuspended in 100 μL of 0.1 ng μL^−1^ pRed. This mixture was transferred to a pre-chilled 2 mm gap electroporation cuvette and electroporated at 2,500 V, 400 Ω, and 25 μF using a Gene Pulser II (Bio-Rad, San Diego, CA, USA). The electroporated cells were immediately recovered in 800 μL SOC and incubated at 30�C for 90 min with vigorous shaking. The whole outgrowth was added to 20 mL of YENB containing 12.5 μg mL^-1^ chloramphenicol and 5 μg mL^-1^ tetracycline and grown overnight at 30�C with vigorous shaking.

After 16 h of growth, 600 μL of culture was used to inoculate 4 mL of fresh YENB containing 12.5 μg mL^-1^ chloramphenicol and 5 μg mL^-1^ tetracycline. This was grown for 3 h at 30�C, or until reaching an OD_600_ >2, at which point 80 μL of 10% l-arabinose was added to induce pRed expression and growth was shifted to 37�C for 1 h with vigorous shaking. About 2 mL of the induced culture was incubated on ice for 2 min, then centrifuged at 5,000*g* for 10 min at 4�C, the supernatant removed, and the pellet placed back on ice. Cells were then washed in 10% glycerol, centrifuged at 5,000*g* for 10 min at 4�C, the supernatant removed, and the pellet placed back on ice. The pellet was resuspended in 100 μL of 5 ng μL^-1^ PCR product and transferred to a pre-chilled 2-mm gap electroporation cuvette, followed by electroporation as before. Electroporated cells were immediately added to 800 μL of SOC and recovered at 37�C for 90 min with vigorous shaking. About 450 μL of outgrowth was spread onto 1.5% LB-agar containing 25 μg mL^-1^ kanamycin, air-dried, and incubated overnight at 37�C. Selected colonies were used to inoculate 4 mL of LB containing 25 μg mL^-1^ kanamycin and grown for 16–18 h at 37�C with shaking. Recombineering products were extracted and validated by restriction digest using the appropriate enzymes, followed by Sanger sequencing using primers designed to amplify the junctions between the pLM099-derived cassette and the target region.

### Statistics

Confidence intervals for Figure�1A were calculated using the Wilson score interval method based on the number of attempted and successfully cloned ATG-Stop amplicons per size category in Mackinder et�al. (2017). Statistical differences in the distribution of sizes and repeat frequencies between successful and unsuccessful PCR and recombineering targets (presented in Figure�3) were assessed using the Mann–Whitney *U* test. A non-parametric test was chosen based on results of the Kolmogorov–Smirnov test for normality for recombineering targets. Test statistics are detailed in Supplemental Table 1.

### Genome analysis

Chlamydomonas, Arabidopsis, yeast, and wheat nuclear genes were analyzed for gene size and sequence complexity. Gene sizes are defined from the start of the 5′-UTR to the end of the 3′-UTR. Note that in Figure�1A, the predicted clonable proportion of genes in each size category is based on cloning success for ATG-Stop regions, not full genes. Sequence complexity is defined in relation to intron prevalence, GC content, and the prevalence of various repeat regions. We designate regions containing a high frequency of repeats as being more complex than regions with a low frequency. This reflects the increased potential for cloning complications presented by sequences with large numbers of repetitive regions, though it differs from descriptions given by Morgulis et�al. (2006). Sequences were analyzed for complexity using the freely available bioinformatics software detailed below (see Supplemental Protocol 3 for settings), and outputs were processed using custom python scripts (see Supplemental Protocol 4 for usage information). GC content was calculated using annotated bases only.

*Sequence data sources*: Unspliced Chlamydomonas nuclear gene sequences used for the analyses were generated using a custom python script (available in the associated GitHub repository) to extract whole-gene, 5′-UTR, ATG-Stop, and 3′-UTR sequences from the genome based on their start and end positions in the current gene models (Phytozome version 5.5). Chlamydomonas gene models are based on predictions using Augustus (annotation version u11.6) and refined using a range of RNA-seq datasets. Files containing the whole genome nucleotide sequence (version 5.0) and the annotation information for each of the 17,741 nuclear genes (version 5.5) were downloaded from Phytozome 12 and are provided as precursor files for running the BACSearcher script (see Supplemental Protocol 2). Sequence data for *A. thaliana* (TAIR10 assembly) and *Triticum aestivum* nuclear genes (International Wheat Genome Consortium assembly) were obtained from EnsemblPlants BioMart. Analysis was limited to the 105,200 chromosome-assigned wheat genes. Sequence data for *Saccharomyces cerevisiae* (S288C reference genome, 2015 release) were obtained from the Saccharomyces Genome Database. Gene sequences were appended to include all annotated UTRs and introns, resulting in a dataset that is more closely comparable to the unspliced gene data used for Chlamydomonas, Arabidopsis, and wheat.

*Analysis of repeats*: Repetitive regions in the nucleotide sequences analyzed in this work are categorized into simple and global repeats. We use the term simple repeats to refer to relatively short (tens to hundreds of bases) repetitive regions in a nucleotide sequence that display regular or semi-regular repeating patterns. We include consecutive repeating motifs of varying unit lengths, known as tandem repeats, as well as inverted patterns in which a short region is followed closely (or immediately, if palindromic) by its reverse complement sequence. Chlamydomonas genes were analyzed for tandem repeats using Tandem Repeats Finder (Benson, 1999). The default settings were modified to provide a cut-off for detection such that no repeats under 10 bp in length were reported (see Supplemental Protocol 3A). All Tandem Repeats Finder outputs were processed using a custom python script and analyzed in spreadsheet format to generate mean values for the number of genes with either: (1) at least one mononucleotide repeat ≥10 bp in length and with ≥90% identity; (2) at least one di- or trinucleotide repeat ≥20 bp in length with ≥90% identity; (3) at least one tandem repeat ≥20 bp in length, with a period length of four or more (tetra+), with ≥90% identity; and (4) the mean number of repeats of these types per kilobase of sequence.

Chlamydomonas genes were analyzed for inverted repeats using the Palindrome Analyser webtool (Br�zda et�al., 2016), available at http://palindromes.ibp.cz/#/en/palindrome. The default settings were modified to report repeats with a maximum of 1 mismatch for every 10 bp of stem sequence, a maximum spacer length of 10 bp, and a maximum total length of 210 bp (see Supplemental Protocol 3B for settings). All Palindrome Analyser outputs were downloaded and analyzed in spreadsheet format to generate mean values for the number of genes containing one or more inverted repeats over 20 bp long with ≥90% identity and the mean number of inverted repeats of this type per kilobase.

All nuclear genes from Chlamydomonas (Figure�1B), Arabidopsis, yeast, and wheat (Figure�1F), and recombineering target regions (Figure�3B and C) were analyzed for global repeats using the NCBI WindowMasker program (Morgulis et�al., 2006). We use the term global repeats to denote the combined number of individual masked regions detected by the WindowMasker modules DUST and WinMask. DUST detects and masks shorter repetitive regions including tandem and inverted repeats, overlapping with and providing support for the Tandem Repeats Finder and Palindrome Analyser outputs. WinMask detects and masks families of longer repetitive regions that do not necessarily occur adjacently in the genome. Default settings were used throughout (see Supplemental Protocol 3C). These modules mask repetitive regions using only the supplied sequence as a template.

Chlamydomonas repeats localized to the 5′-UTRs, ATG-Stop regions, and 3′-UTRs were distinguished using positional information from Phytozome (genome annotation version 5.5). Repeats that spanned from a 5′-UTR across the start codon or across the stop codon into the 3′-UTR were not counted, although were included in the whole-gene repeat analyses described above.

*uORFs, transcripts and intron analysis*: Data on the presence of uORFs in Chlamydomonas transcripts were obtained from the results of a BLASTP analysis performed by Cross (2015) and adapted to provide the per-gene values. A list of Chlamydomonas transcripts was downloaded from Phytozome Biomart and used to identify the number of genes with more than one transcript model. Genomic data detailing the number and order of exons within each gene were also downloaded from Phytozome Biomart; this information was used to ascertain the number of genes containing introns in their translated and untranslated regions.

*Primer analysis*: To assess the impact of inefficient priming on PCR-based cloning, analysis was performed on a dataset of PCR primers designed to clone every gene in the Chlamydomonas genome from start to stop codon using gDNA as the template and generated such that the predicted *T*_m_ difference for each pair was not more than 5�C where possible. Primer sequences were then assessed against four thresholds pertaining to efficient priming, set in accordance with advice found in the Primer3 manual, support pages provided by IDT, and the Premier Biosoft technical notes. These thresholds relate to primer length, propensity for secondary structure formation, the presence of repeats, and the GC content of the 3′-end. Long primers can have a reduced amplification efficiency, secondary structure formation can reduce the number of primers available to bind to the intended template during a PCR, multiple repeats can increase the risk of mispriming, and a high 3′-end GC content can increase the risk of primer–dimer formation. Thresholds for each were set as follows: (1) primer length should not be more than 30 bp; (2) the Δ*G* required to disrupt predicted secondary structures should be above −9 kcal mol^-1^ at 66 or 72�C; (3) tandem single nucleotides or dinucleotide motifs should repeat no more than 4 times, and (4) the 3′-end should consist of no more than 4 G/C bases in a row. The number of primers in breach of each of these thresholds is shown in Figure�1D as a percentage of the dataset. The percentage of unsuitable primer pairs was calculated by counting pairs for which one or both primers breached one or more of these thresholds. *T*_m_ considerations were omitted from analysis since Chlamydomonas genes have an unusually high GC content, so primers designed to amplify gDNA are expected to have higher than recommended *T*_m_’s according to generic primer design guidelines. GC content was calculated using annotated bases only.

To complement these results, primers were analyzed using the check_primers algorithm from Primer3 (Rozen and Skaletsky, 2000). The settings used were the default settings for Primer3Plus (Untergasser et�al., 2007)—an updated, online version of the Primer3 package—with minimal modifications that included removing the Tm constraints (see Supplemental Protocol 3D for full settings used). The output was analyzed with a custom python script that reported the primary reason for rejection of individual primers (see Supplemental Protocol 4C). *T*_m_ was removed as a constraint to allow for more detailed analysis of primer sequence parameters, since the default maximum allowable *T*_m_ for Primer3Plus is 63�C, which results in rejection of almost 90% of primers for this reason alone if used. 1.6% of primers were too long to be considered for analysis (>36 bp); these were included in Figure�1D (orange bar) as having been rejected for breaching the length constraint. The majority of rejected primers produced one of the following three reasons for rejection: (1) “high end complementarity” for primer pairs, which implies a high likelihood that the 3′-ends of the forward and reverse primers will anneal, enabling amplification of a short, heterogeneous primer-dimer (cross-dimer); (2) “high end complementarity” for single primers, which implies a high likelihood that a primer’s 3′-end will bind to that of another identical copy, self-priming to form a homogenous primer-dimer (self-dimer); and (3) “high any complementarity” for single primers, which implies a high likelihood of self-annealing without necessarily self-priming, relevant to both the inter-molecular annealing of identical copies and to instances of hairpin formation resulting from intra-molecular annealing. Primers rejected for these three reasons are labeled in Figure�1D (orange bar) as cross-dimers, self-dimers, and hairpins, respectively.

*Note on differences between the Chlamydomonas BAC library strain and CLiP mutant strain*: The Chlamydomonas BAC library was constructed using the genome reference strain CC-503, so researchers working with alternative strains need to take into account potential genomic divergence. For example, here we transformed recombineered DNA from the BAC library into CC-4533, the wild-type strain used for the CLiP mutant collection and a popular strain for studying the CCM. Genomic analysis of CC-4533 relative to CC-503 has revealed 653 instances of variation that may be disruptive to protein function, although only three of these are unique to CC-4533 compared to other common laboratory strains (Li et�al., 2016). Two genes affected by this variation were successfully cloned using our recombineering pipeline: Cre06.g250650 in CC-4533 contains three short deletions relative to CC-503 with an uncertain impact on the protein, while Cre06.g249750 in CC-4533 contains a predicted inversion affecting the final three exons and part of the 3′-UTR.

### BACSearcher python resource

Suitable BACs containing the target genes were identified using a python script that also identifies 50-bp binding sites for recombineering cloning primers and provides sequences for primers that can be used to check for the presence of a target gene within a BAC (see Supplemental Protocol 2). BACSearcher output is available for all 17,741 genes in the genome in Supplemental Data Set 1. For individual targets in our recombineering pipeline that were not covered by a BAC in the BACSearcher output, an alternative method was employed to search for BAC coverage. This method is detailed in Supplemental Protocol 2, along with usage and modification instructions for BACSearcher, including instructions on how to output suitable fosmids for all genes in the genome. BACSearcher resources can be found in the associated GitHub repository at https://github.com/TZEmrichMills/Chlamydomonas_recombineering.

### Accession numbers

Sequence data from this article can be found in Phytozome, the Plant Comparative Genomics portal of the Department of Energy's Joint Genome Institute, under the following accession numbers: Cre11.g467712: SAGA1; Cre09.g412100: PSAF; Cre03.g155001: ISA1; Cre10.g435800: CSP41B; Cre17.g702500: TAB2; Cre10.g452800: LCIB; Cre09.g394473: LCI9.

All plasmid sequences are available in Supplemental Data Set 4 and have been deposited in GenBank with the following IDs: pLM099, MT737960; pLM160, MT737961; pLM161, MT737962; pLM162, MT737963; pLM459, MT737964. Plasmids are available from the Chlamydomonas Resource Center (https://www.chlamycollection.org/), as are the BAC and fosmid libraries. Full protocols for batch and high-throughput recombineering are available in Supplemental Protocol 1. Data used for the genome analyses presented in Figure�1 are available on request. The python computer code used for identifying BACs, fosmids and suitable homology regions for recombineering is available at https://github.com/TZEmrichMills/Chlamydomonas_recombineering.

## Supplemental Data

The following materials are available in the online version of this article.

**Supplemental Figure 1.** Batch-scale recombineering results.

**Supplemental Figure 2.** Validation of fluorescently localized lines.

**Supplemental Figure 3.** Complementation of the *lcib* CLiP mutant.

**Supplemental Table 1.** Mann-Whitney U test statistics.

**Supplemental Protocol 1.** Protocols for batch and large-scale recombineering.

**Supplemental Protocol 2**. BACSearcher usage.

**Supplemental Protocol 3.** Bioinformatics software usage.

**Supplemental Protocol 4.** Bioinformatics python analysis.

**Supplemental Data Set 1.** BACSearcher output.

**Supplemental Data Set 2.** Large-scale pipeline results summary.

**Supplemental Data Set 3.** Oligonucleotide sequences.

**Supplemental Data Set 4.** Plasmid sequences.

## Supplementary Material

koab024_Supplementary_Data

## Notes

T.Z.E.M. developed the initial recombineering pipeline, designed and assembled the original pLM099 recombineering plasmid and performed the genome wide analysis. G.Y., T.Z.E.M., and T.K.K. assembled additional recombineering plasmids. T.Z.E.M. and G.Y. optimized and performed the large-scale recombineering pipeline. G.Y. performed the microscopy and Venus quantification data. P.G. validated the pipeline using fosmids. J.B. performed the complementation experiments. J.B., I.G., C.S.L., C.E.W., and T.K.K. supported the development and implementation of the recombineering pipeline. J.W.D. wrote the BACSearcher code and provided bioinformatics support to T.Z.E.M. for the remaining code. L.C.M.M. conceived the idea and led the research. L.C.M.M. and M.P.J. received funding to support the work. L.C.M.M., T.Z.E.M., and G.Y. wrote the manuscript.

The author responsible for distribution of materials integral to the findings presented in this article in accordance with the policy described in the Instruction for Authors (https://academic.oup.com/plcell) is Luke C.M. Mackinder (luke.mackinder@york.ac.uk).
