# Supplementary material for: A recombineering pipeline to clone large and complex genes in Chlamydomonas
Source: Plant Cell. 2021 Feb 2;33(4):1161–81. doi: 10.1093/plcell/koab024 (PMC8633747; doi:10.1093/plcell/koab024)
Supplement: koab024_Supplementary_Data [file koab024_supplementary_data.zip › tpc.00363.2020-s03.pdf]

Supplemental Figure 1. Batch-scale recombineering results

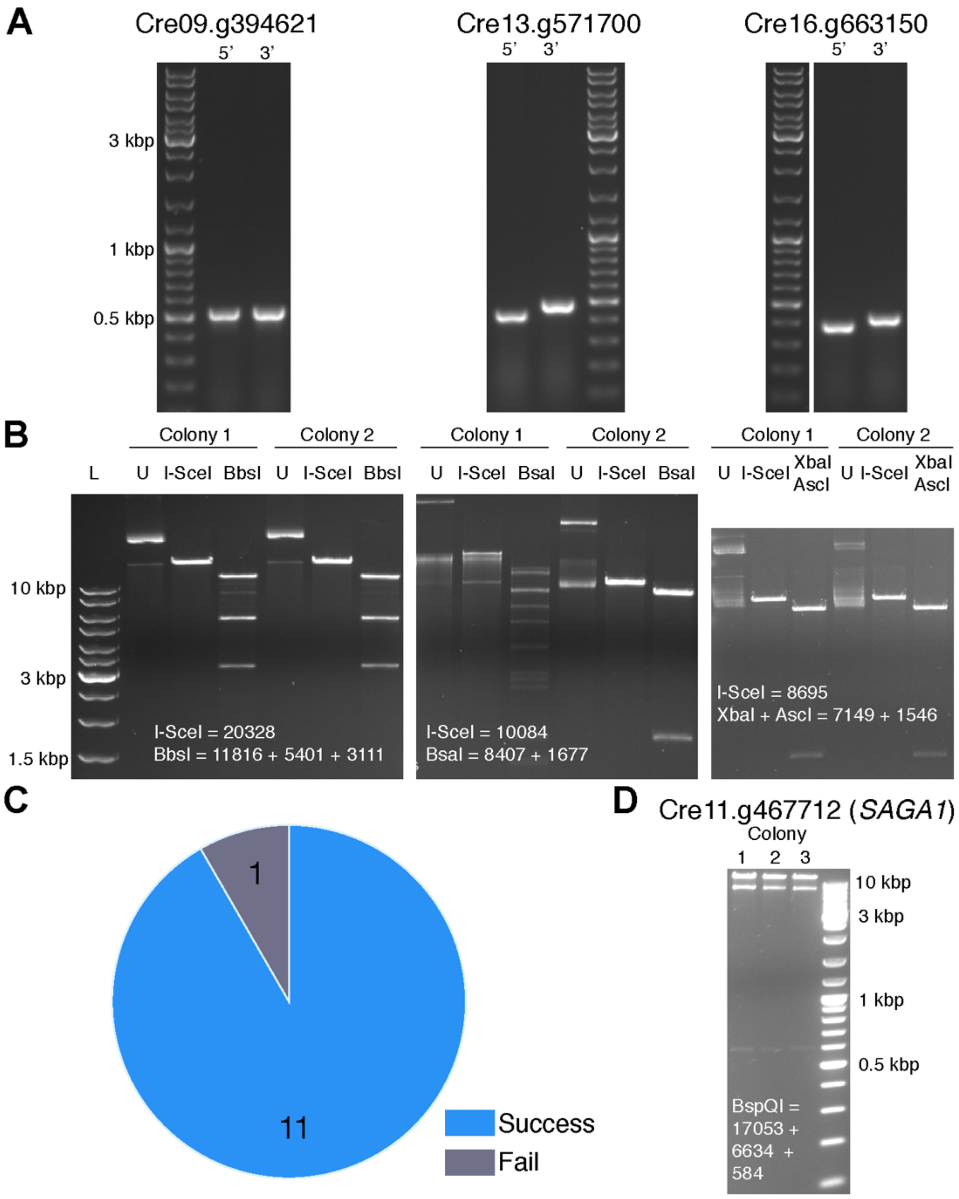

Supplemental Figure 1. Batch-scale recombineering results. (Supports Figure 2.)
A Three examples of colony PCRs to check for presence of target genes in BACs. Primer pairs were designed to the 5' and 3' end of each target gene. All amplicons were of the expected size.
B Restriction digest checks for isolated recombineered plasmids from two colonies per gene, corresponding to the same genes as in **A**. Expected sizes are shown in bp. Note that colonies 1 and 2 for Cre09.g394621 produced low-abundance bands in addition to the expected banding pattern that potentially correspond to incomplete digestion products. Colony 1 for Cre13.g571700 gave the incorrect size and banding patterns after digestion indicating incorrect recombination. L: GeneRuler 1 kb DNA Ladder (ThermoFisher Scientific). U: undigested.
C Overall batch-scale recombineering success for 12 target genes.
D Restriction digest checks of plasmids isolated from three colonies for *SAGA1* recombineered from fosmid VTP41289 using pLM160.

Supplemental Figure 2. Validation of fluorescently localised lines

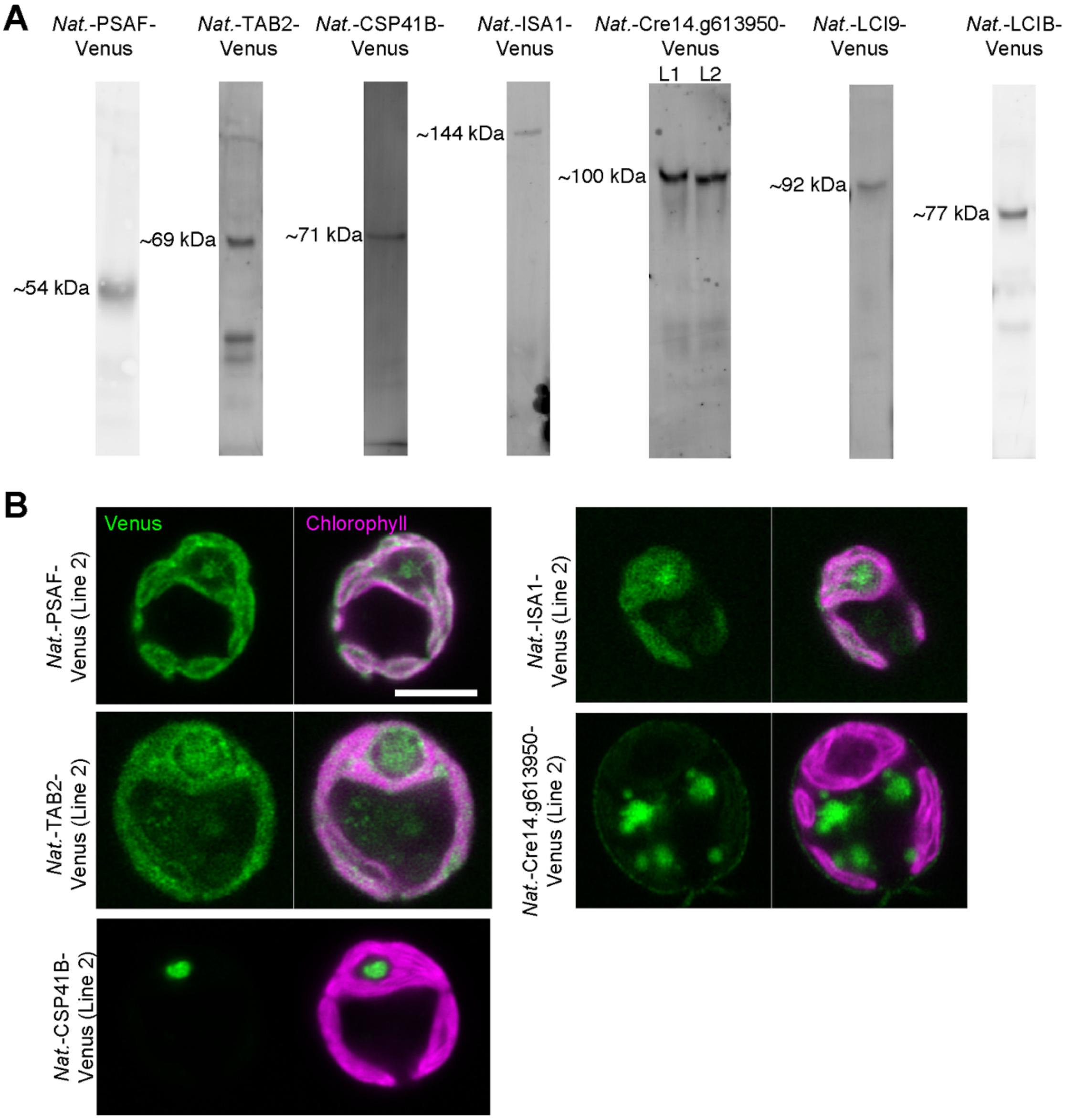

Supplemental Figure 2. Validation of fluorescently localized lines. (Supports Figures 4 and 5.)

A Immunoblots against the 3xFLAG epitope for recombineered targets. Molecular weights indicate the approximate band size. All cloned targets except Cre14.g613950 (expected molecular weight of 141 kDa) showed the expected molecular weight. Two independent transformants were tested for Cre14.g613950 to confirm that the observed lower molecular weight was consistent between transformants. Contrast/brightness were adjusted separately for each image.

B Localization of target proteins in additional independent transformants (line 2). All localizations are consistent with line 1 localizations shown in Figure 4. Scale bar: 5 µm.

Supplemental Figure 3. Complementation of the *lcib* CLiP mutant

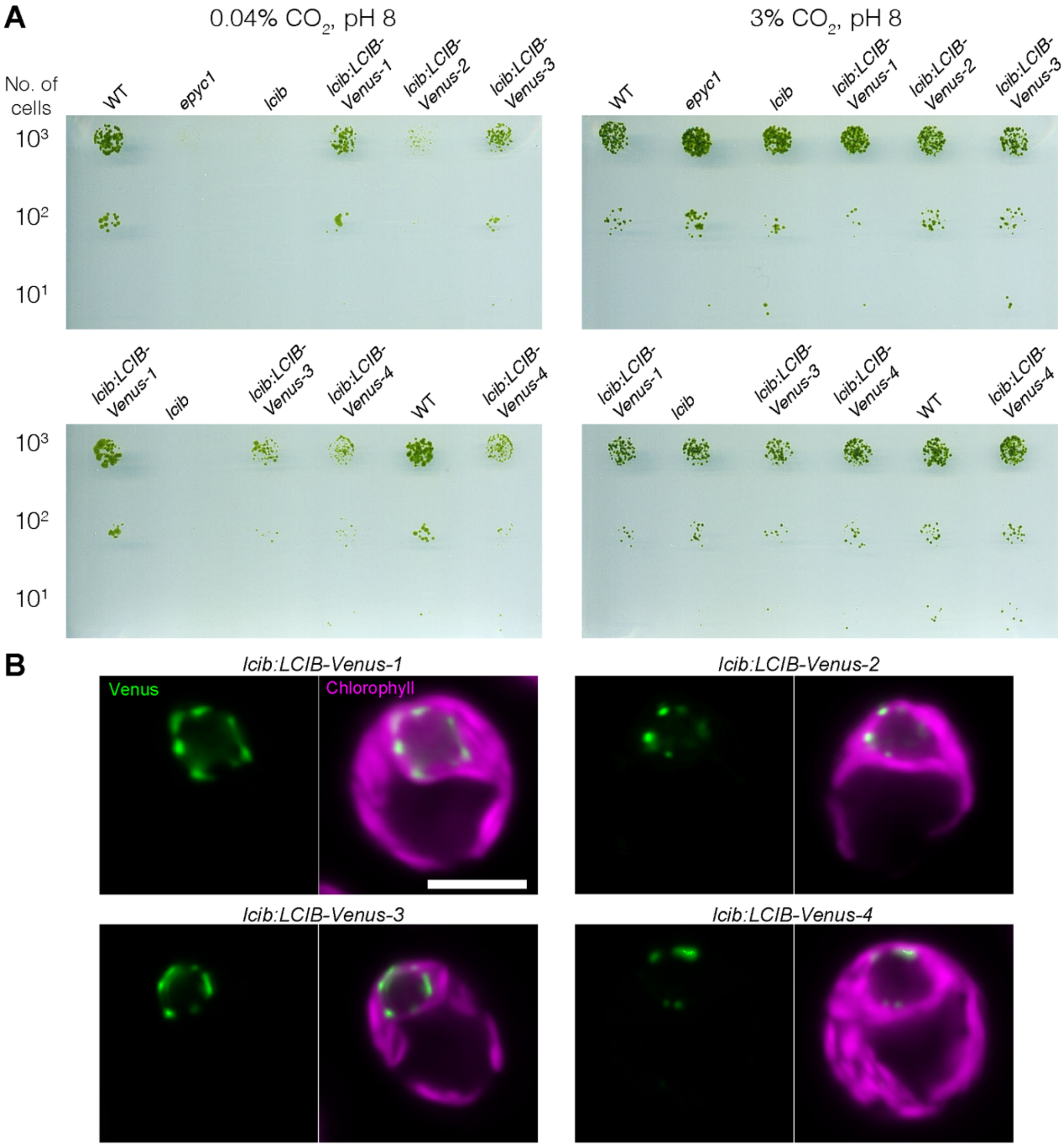

Supplemental Figure 3. Complementation of the *lcib* CLiP mutant. (Supports Figure 5.)
A Spot tests of *lcib* CLiP mutant LMJ.RY0402.215132 complemented with recombineered *LCIB-Venus* driven by its native promoter. Four independent transformants were spotted onto pH 8 TP minimal media plates and grown at 0.04% and 3% CO_2. The *epyc1* mutant that has a severe CCM phenotype due to incorrect pyrenoid assembly was included as a CCM growth phenotype control. Note varying degrees of complementation between lines. Top and bottom images for each CO_2 condition are from the same plate but split for labelling clarity.
B Corresponding confocal microscope images of complemented lines all showing the typical localization of LCIB at the pyrenoid periphery. Scale bar: 5 µm.

Supplemental Table 1. Mann-Whitney U test statistics.

Supplemental Table 1. (Supports Figure 3.) Test statistics for the Mann-Whitney U test applied to differences in the distribution of region sizes and frequency of repeats (repeats detected by WindowMasker per kilobase) for successfully/unsuccessfully cloned PCR and recombineering targets. All tests were two-tailed.

Size (bp)	Successful mean/median (N)	Unsuccessful mean/median (N)	*U*-value	*Z*-score	*p*-value
ATG-Stop PCR (Mackinder *et al.* 2017)	2313/2141 (298)	4485/4210 (326)	16306	14.30	<0.001
Recombineering (this study)	7315/6213 (157)	7416/5873 (46)	3303	0.878	0.379

Repeat frequency	Successful mean/median (N)	Unsuccessful mean/median (N)	*U*-value	*Z*-score	*p*-value
ATG-Stop PCR (Mackinder *et al.* 2017)	6.067/5.903 (298)	7.540/7.646 (326)	24110	10.56	<0.001
Recombineering (this study)	6.396/6.485 (157)	6.790/6.999 (46)	3129	-1.376	0.167

References

Mackinder, L.C.M., Chen, C., Leib, R.D., Patena, W., Blum, S.R., Rodman, M., Ramundo, S., Adams, C.M., and Jonikas, M.C. (2017). A Spatial Interactome Reveals the Protein Organization of the Algal CO_2-Concentrating Mechanism. Cell **171,** 133-147.e114.
