# Supplementary material for: A recombineering pipeline to clone large and complex genes in Chlamydomonas
Source: Plant Cell. 2021 Feb 2;33(4):1161–81. doi: 10.1093/plcell/koab024 (PMC8633747; doi:10.1093/plcell/koab024)
Supplement: koab024_Supplementary_Data [file koab024_supplementary_data.zip › tpc.00363.2020-s05.pdf]

A Recombineering Pipeline to Clone Large and Complex Genes in Chlamydomonas

Tom Z. Emrich-Mills, Gary Yates, James Barrett, Philipp Girr, Irina Grouneva, Chun Sing Lau, Charlotte E Walker, Tsz Kam Kwok, John W. Davey, Matthew P. Johnson, Luke C.M. Mackinder

Corresponding author: Luke C.M. Mackinder (luke.mackinder@york.ac.uk).

Review timeline:

**TPC2020-LSB-00363**	Submission received:	May 7, 2020
	1st Decision:	June 11, 2020 *revision requested*
**TPC2020-LSB-003631**	1st Revision received:	Nov. 17, 2020
	2nd Decision:	Dec. 9, 2020 *accept with minor revision*
**TPC2020-LSB-00363R2**	2nd Revision received:	Dec. 18, 2020
	3rd Decision:	Dec. 18, 2020 *acceptance pending, sent to science editor*
	Final acceptance:	Jan. 8, 2021

REPORT: (The report shows the major requests for revision and author responses. Minor comments for revision and miscellaneous correspondence are not included. The original format may not be reflected in this compilation, but the reviewer comments and author responses are not edited, except to correct minor typographical or spelling errors that could be a source of ambiguity.)

TPC2020-LSB-00363 1st Editorial decision – *revision requested* **June 11, 2020**

The description of the recombineering pipeline must be improved, by providing additional details on the tools developed. This will be important to make the methodology accessible and attractive also for the algal and plant communities beyond Chlamydomonas and Arabidopsis. Useful suggestions for improving the Introduction and Results sections have been made by the three Reviewers. The authors may also consider moving Figure S1 into the main manuscript. Additional details are also required for the genomic analyses shown in Figure 1 and the BAC libraries used in this study. The Editors agree with Reviewer 3 that it will be very important to provide additional details about the ability of individuals to get access to the protocols, plasmids and software.

The Editors concur with Reviewers 1 and 3 that additional analyses are needed to support the functional application of different recombineering vectors. In particular:
(i) you should show by immunoblots that the fusion proteins generated with the recombineering strategy have the expected size. This is important to exclude the presence of an internal start or perhaps a proteolytic cleavage site.
(ii) you should demonstrate that the fusion proteins are functional. This can be done by performing the localization analysis in a mutant background and showing rescue of the mutant phenotype.
(ii) the analysis of the localization of the LCIB protein as shown in Figure 5 by using 2 different promoters (native vs. PsaD) is insufficient to justify the generalized conclusions. You should extend the analysis to other genes.

RESPONSE: We thank the editors and reviewers for the constructive comments and suggestions for the further improvement of the manuscript. We have addressed all comments below. In addition to addressing the reviewers' comments, we have since tested our recombineering pipeline for cloning genes from fosmids. We show that the pipeline can be successfully used for fosmids and have now included this in the Results (lines 342-353) and provide data as panel Figure S1D. We believe this is a considerable further addition to the manuscript, as the newly available fosmid library from the Chlamydomonas Resource Center has close to complete coverage of the genome, thus now enabling nearly all gene targets to be cloned via our recombineering pipeline. Related to this, we have added Philipp Girr to the author list who validated the pipeline for fosmids.

We have added additional details of the developed pipeline, available tools and resources. We have also incorporated the reviewer suggestions for improving the Introduction and Results sections. Changes are highlighted in the individual responses to each Reviewer and also include the following:

At the start of the Methods we have added a section "Availability of materials data and software" (lines 651-659) that provide a reference to the Supplementary Data that includes a detailed step-by-step protocol. This section also includes links to all reagents and code.

We have added further details on how the developed recombineering framework could be easily adopted for other organisms with a BAC or fosmid library (lines 616-622).

We have highlighted in the "Recombineering pipeline development" section how the pipeline can be used to clone genes without a fluorescent tag or with a small affinity tag (lines 330-332).

We have moved Figure S1 to Figure 5 panel A. The previous Figure S2 is now Figure S1.

We have added additional details in the results section "Analysis of the Chlamydomonas genome highlights the challenges affecting PCR-based cloning" (lines 154-217), in the Figure 1 legend and the "Genome analysis" section of the Methods (lines 788-864). All changes are tracked.

Additional information on the BAC library has been included in the "Availability of materials, data and software" section (lines 654-655) and a brief discussion of the strain variability between the BAC source strain (CC-503) and the line used for expression of recombineered constructs (CC-4533) has been added to the Methods (lines 925-944).

A key driving force for this work was to make all reagents and protocols easily accessible to the field. To do this we have:

Added a subsection to the Methods, "Availability of materials, data and software" that clearly list all sources for resources, data and software (lines 651-659).

Added link to CRC website (line 655).

Deposited all recombineering plasmids to CRC with descriptions.

Uploaded plasmid sequences to GenBank and added IDs into manuscript (lines 652-654).

We have performed immunoblotting against the 3xFLAG epitope for the five recombineered fluorescently tagged lines shown in Figure 4 plus LCI9-Venus and LCIB-Venus shown in Figure 5. We have included this as Figure S2.

Interestingly, for Cre14.g613950, we detected a lower than expected band in two independent transformants at ~100 kDa (expected ~141 kDa). Re-analysis of the available gene model shows a run of 2,615 "NNN" within intron 14 starting at position 6,515 of the gene model. No transcripts mapped to Cre14.g613950 spanned across this intron. We propose that the current gene model maybe incorrect or that the translated protein undergoes post-translational cleavage prior to/during transport to the plasma membrane. We have added the following to lines 490-494: "However, immunoblotting against the C-terminal 3xFLAG tag of Cre14.g613950 in two independent transformants shows a smaller molecular weight band than predicted (Figure S2). This potentially indicates that the gene model for Cre14.g613950 is incorrect or that the protein undergoes post-translation cleavage as seen for other CCM related proteins that transit via the secretory pathway (Fukuzawa et al., 1990; Tachiki et al., 1992)."

We have now shown an example of where the fusion proteins can be used for complementation of a mutant from the Chlamydomonas Library Project, which indicates localization is unaffected. For this we used the *Native-LCIB-Venus* construct to complement the *lcib* mutant. We show this in Figure S3. For more information and a detailed response to this point please, see response to point 5 raised by Reviewer #3.

--- Reviewer comments:

Reviewer #1 (Comments for the Author):

In this manuscript, Emrich-Mills et al. describe the development of a recombineering pipeline for the cloning of large and complex Chlamydomonas genes. The pipeline takes advantage of an existing BAC library covering about 86% of the Chlamydomonas nuclear genes. It allows the recombination of a region of choice of up to 23 kb out of the BAC into a destination vector that is equipped with several different fluorescing proteins, the PSAD terminator, and three different selection markers for Chlamydomonas. Upon transformation into Chlamydomonas, the target gene is then expressed from its native promoter (~2 kb upstream of start codon) with the fluorescent protein fused to the C-terminus. The authors tested the system with 191 target genes and were successful with 146 (76% success rate). They demonstrate that the cloning success is independent of the length of the cloned region, and of repeats within.

They show the localization of five selected targets that have previously been shown to be associated with the carbon concentrating mechanism. Finally, they show that the localization of LCIB to the periphery of the pyrenoid was the same when the LCIB gene was driven by its own promoter or the PSAD promoter.

The manuscript is extremely well written and presented, it was a pleasure to read. There are only few issues that the authors need to address (see below). Since all biological data shown are confirmative, the main audience of this work will be Chlamydomonas researchers that are struggling with the cloning of large, complex genes, i.e., an audience limited to only a part of the Chlamydomonas community. While this pipeline will be of greatest value for this audience, I am afraid that it will be of less interest to researchers working on land plants, for which a similar pipeline has been published recently (Brumos et al., 2020), or other algal systems, to which this system is not readily transferable.

Specific points:
1. Introduction, end of second paragraph: it is not necessary to synthesize the full gene, smaller pieces can be synthesized as gene blocks and assembled by Golden Gate cloning. Since the costs for gene synthesis are steadily decreasing, this is likely the method of choice in future.
2. Page 6, top: how sure is it that the very large genes in Chlamydomonas are correctly annotated? Perhaps this can be addressed by comparison with genes in Volvox and Gonium?
3. I am missing a confirmation that the localization of the five example genes in Figure 4 is not the result of the in-frame integration of a broken construct into another gene. This could be addressed by providing information on how many independent transformants gave the same localization results, RT-PCR, or proteomics on a YFP pull-down.
4. Figure 5D: How many independent transformants have been tested here? This information is important, as the expression could be driven by another promoter, if the construct got disrupted and integrated into another gene (promoter trap). In this line, the authors show expression results only for a single gene, LCIB. Is this enough to extrapolate to all other promoters?
5. The authors mentioned that "57 of the 298 successfully cloned genes from Mackinder et al. (2017) contained a class 1 in-frame ATG upstream of the cloned region, therefore ~10% of cloned regions may have encoded truncated protein products". It would have been interesting to revisit these cases with genes cloned with the pipeline presented here to verify correct localization.

Reviewer #2 (Comments for the Author):

The manuscript by Luke Mackinder and colleagues reports the development of a recombineering pipeline to clone large genes in the green unicellular alga *Chlamydomonas reinhardtii*. The authors developed a method enabling parallel cloning of genes through homologous recombination in *E. coli* from the Chlamydomonas BAC collection. The method was tested on 191 genes of the CO2 concentrating mechanism with an overall success rate of 77%. The authors show that unlike PCR-based methods, the success rate of the recombineering strategy is independent of gene size. In addition, the authors developed a series of vector tools that enable complementation of the CLIP collection of insertional mutants and diverse tagging strategies for subcellular localization, immunodetection and affinity purification of target proteins. These tools were validated and allowed to confirm the subcellular localization of diverse proteins. Finally, the authors developed a software tool enabling the design of recombineering sequences for any Chlamydomonas gene, they provide a dataset including sequences for the top five optimal 5' and 3' homology regions for each target gene in the genome and detailed protocols.

The manuscript is very clearly and concisely written. The recombineering pipeline developed and the tools, software and dataset provided are important contributions that will be of great interest for the whole Chlamydomonas community. Some suggestions and comments to improve the manuscript are listed below.
- A major concern for the development of synthetic biology tools is to standardize the tools to enable modularity. Despite the clear interest of the recombineering pipeline presented, I regret that it lacks modularity. It would have been interesting to develop tools compatible with other standardized tools developed for Chlamydomonas, such as the Chlamydomonas Moclo Toolkit (Crozet et al. 2018, ACS Synth Biol). The nuclear sequences obtained through recombination with BAC would not be standardized (i.e. not domesticated due to the presence of undesired restriction sites) but standardization could have been used to design the recombineering vector tools in order to enable compatibility with all the MoClo bricks previously developed. This would broaden the possibilities of the

pipeline by enabling the use of alternative bricks including alternative promoters, antibiotic resistances or tags.
- The beginning of the Results section underscores problems in cloning Chlamydomonas genes but assumes that the only way to do so is through a PCR starting from the annotated ATG and ending at the annotated STOP. One can perfectly clone the entire genomic region through assembly of multiple parts generated by PCR or gene synthesis. For example, Golden Gate cloning can be a powerful tool to assemble scarlessly in one step multiple fragments obtained by PCR or synthesis. These points could be discussed in the manuscript.
- The genomic analyses provided in the Results section are quite interesting but lack precision on how the data were generated or where they come from. For instance, are the position of introns based on gene models or were some introns/gene models modified (e.g. annotated from cDNA/RNAseq analysis)? This should be more clearly stated.
- Discussion - p10 - line 6: The authors indicate in the Discussion that "In addition, all vectors can be used for cloning genes without fluorescence tags or with just short affinity tags (3xFLAG and 3xHA)". This point is important and should be more clearly stated in the results section.
- The authors should clarify the usage of some words. For example, the term "gene" refers to 1/ the genomic intron-containing sequence encompassing the promoter, the coding sequence and UTRs (section in 'recombineering pipeline development') or 2/ only the transcribed regions (for instance in fig1F) or 3/ only the coding sequence (section 'gene complexity'). This should be corrected.
- Results - gene complexity - line 10: "Of the 17,741 coding genes in the genome" could be replaced for clarity by "Of the 17,741 coding genes in the nuclear genome".
- Fig S1: this figure gives a nice overview of the tools developed for the recombineering strategy. I think this figure is important and should be included as a main figure.
- Fig S2B: An extra band in colony 1 digestion with Bbs1 of Cre9.g394621 is not discussed.
- Fig 5 The authors should clarify whether "PSAD promoter" refers to the promoter including its 5' UTR.
- Fig 1C: The authors should indicate that more than one uORF can be found in each gene. In addition, showing the intersection between each category (including splice variants, 3'UTR introns, etc.) would be interesting.

Reviewer #3 (Comments for the Author):

This is a techniques paper that focuses on a pipeline for cloning, through recombineering, specific genes from the Chlamydomonas BAC collection. It in part provides a summary of what makes PCR-based cloning fail in Chlamydomonas, a useful and more organized summary than previously presented by others... providing valuable cautionary information for researchers cloning genes from this alga. Large and complex genes can be cloned using this recombineering approach, many of which cannot be readily cloned by PCR based methods. The authors used this technique to clone genes associated with the Chlamydomonas CCM, achieving a success rate of 77% (using a pool of 191 genes). These cloned genes are being used to localize gene products within Chlamydomonas cells, rescue mutant strains and identify new pyrenoid components (through fusions with sequences encoding various of fluorophores). The recombineering cassette can also include sequences that target the encoded fusion proteins to specific compartments in the Chlamydomonas cell, and can facilitate the evaluation of protein levels (e.g. exposure of cells to different conditions) and interactions. This is a technology that can be useful for the cloning of 'recalcitrant' genes (of which there are many in Chlamydomonas), which would expand the tools available to explore gene function.

The work seems to be generally well done and provides an additional tool that will benefit the Chlamydomonas community, especially when researchers are dealing with very large genes and/or genes with various repetitive elements. The rationales for using this recombineering approach are sound and well documented. While the text makes various arguments concerning the utility of this procedure. I think that parts of the text can be tightened (Introduction) while some aspects of the results can be expanded to give the reader additional useful and interesting information (repeat element information, see below).
1. It might be better to condense the Introduction; it is a little repetitious and sometimes contains more detail about the CCM and the methods than is necessary. This applies to the abstract and Results to some extent as well... for example, is the sentence 'We initially applied the pipeline to 12 targets with a 92% cloning success rate' necessary in the abstract. In the results under 'Genetic complexity' is the first sentence necessary? There are additional places that would benefit from some pruning.

2. The Introduction discusses the 2017 Mackinder papers in which an attempt was made to clone 624 gDNAs encoding proteins with putative CCM functions (successfully cloning 298). The relationship between the 191 genes used to test the recombineering method and the original 624 genes is not absolutely clear. Did the current test population only contain genes that were not successfully cloned by the PCR based approach (we are told it is 'primarily' from the CCM gene population examined in 2017)? Is there any particular reason why these genes were chosen (rather than using all 326 genes not successfully cloned in the earlier work; or were 135 of the 326 genes not represented in the BAC library)? I am not sure that the sentence 'For the 146 correctly recombineered lines, picking just a single colony gave a 63% success rate, screening a second colony increased the success rate to 85% and a third colony gave a 97% success rate, for a small proportion of targets screening >3 colonies led to the identification of a correctly recombined construct (Figure 2E)' is structured properly. I am assuming that the 14 cases in which a different BAC (harboring the same target gene) had to be chosen to make the procedure work were part of the 146 that worked (so initially 132 worked with 14 additional successes when another BAC was used?). The numbers are a little confusing; in the Introduction it says that the success in cloning the genes was 77%, which is ~146 genes, which is what it indicates in the results. However, toward the end of the Discussion it says 157 CCM genes were successfully cloned.
3. The BAC library is from CC-503 while the strain used for transformation is CC-4533. Are there any difficulties that should be considered when using these different resources? For example, is there information indicating that the two strains show identical acclimation responses (same CCM genes upregulated)? Also, unless I missed it on the website, it would be worth providing the Resource Center with a protocol for the recombineering (emphasizing critical aspects, including the growth of the bacteria for transformation with the recombineering cassette), availability to the plasmids used and access to important software, such as BACSearcher (everything to make it easy for most labs to effectively use the technique).
4. It might be worth providing a little more information about the repeats, which I am sure can be readily determined. What is the distribution of mono, di, tri, poly, tandem, inverted repeats in the different domains of the genes (coding region, intron, exon, UTRs)? Are the inverted repeats, which appear in most genes, primarily in the 3' ends? Is there any correlation between the position in the gene and type of repeat? Is there a stronger correlation between cloneability with the type/position of the repeats than just with the presence of repeats? This might provide more information about both the structure of the genome and the factors that impact the ability to clone a gene.
5. Some examples are given for validation of the localization of the fluorescence tagged proteins, although it hasn't been done with the detail needed to absolutely confirm proper localization. It is likely that PSAF is properly localized (at least to the thylakoid membranes), but the introduced PSAF was not shown to rescue a mutant strain or shown to be associated with PSI particles; it could be in thylakoids and yet not be properly localized or functionally competent. The same types of arguments can be made for the TAB2 and Cre14.g613950 and Cre10.g435800. To discuss the validity of the localization, it would be best to show that the fusion proteins rescue the mutant strains, locate to a particular compartment/complex in those strains, and then show that the localization is exactly the same in wild type cells. I am suggesting a little more caution in interpreting the localization data if there is really no check that the protein is functioning properly in the transformants... although I do agree that given prior observations the conclusions are reasonable.
6. In the section of the results on 'Maintaining the native promoter....', the way it is written may be improved a bit. For example, the first sentence in the paragraph 'As our pipeline retains the native promoter of the target gene we hypothesized that fluorescence output would be representative of relative protein abundance' seems a little confusing. Even if the target gene didn't have the native promoter you might hypothesize that the fluorescence output would represent the relative protein abundance. I guess the authors are basically saying that constructs with the native promoter and a fluorophore fusion could be used to monitor protein accumulation (e.g. potentially reflecting changes in gene expression) that occur when environmental conditions are changed, such as changes in expression of genes encoding CCM proteins.
7. Is it valid to imply that you can reach a 92% success rate (in Discussion) when you only tested 12 samples to get this percentage??
8. The meaning of Figure 1A is a little difficult for me to understand... for example, if there were only a few genes of the 624 genes that were in the >10000 category and they all exhibited cloning failure, would that mean that all 1500-

2000 genes in the category would be considered not cloneable? Is there a statistical way to provide a confidence value for this data? I can't tell from the figure, but based on the legend of Figure 1B, there appear to be at least some genes that don't have some form of repeat structure; what are they? Has the structure of the Cre08.g379800 gene been verified by a cDNA sequence?

TPC2020-LSB-00363R1 1st Revision received Nov. 17, 2020

Reviewer comments and **author responses**:

Reviewer #1:

In this manuscript, Emrich-Mills et al. describe the development of a recombineering pipeline for the cloning of large and complex Chlamydomonas genes. The pipeline takes advantage of an existing BAC library covering about 86% of the Chlamydomonas nuclear genes. It allows the recombination of a region of choice of up to 23 kb out of the BAC into a destination vector that is equipped with several different fluorescing proteins, the PSAD terminator, and three different selection markers for Chlamydomonas. Upon transformation into Chlamydomonas, the target gene is then expressed from its native promoter (~2 kb upstream of start codon) with the fluorescent protein fused to the C-terminus. The authors tested the system with 191 target genes and were successful with 146 (76% success rate). They demonstrate that the cloning success is independent of the length of the cloned region, and of repeats within. They show the localization of five selected targets that have previously been shown to be associated with the carbon concentrating mechanism. Finally, they show that the localization of LCIB to the periphery of the pyrenoid was the same when the LCIB gene was driven by its own promoter or the PSAD promoter.

The manuscript is extremely well written and presented, it was a pleasure to read. There are only few issues that the authors need to address (see below). Since all biological data shown are confirmative, the main audience of this work will be Chlamydomonas researchers that are struggling with the cloning of large, complex genes, i.e., an audience limited to only a part of the Chlamydomonas community. While this pipeline will be of greatest value for this audience, I am afraid that it will be of less interest to researchers working on land plants, for which a similar pipeline has been published recently (Brumos et al., 2020)), or other algal systems, to which this system is not readily transferable.

RESPONSE: We would like to thank the reviewer for their positive and supportive comments. Whilst we agree that the method will be most relevant to the Chlamydomonas research community, we think that the approach and design framework would allow rapid conversion of the pipeline for other algal and plant species where a BAC/fosmid library is available (i.e. *Gonium pectorale*). We have now added a comment related to this in the Discussion (lines 615-622).

Point 1. Introduction, end of second paragraph: it is not necessary to synthesize the full gene, smaller pieces can be synthesized as gene blocks and assembled by Golden Gate cloning. Since the costs for gene synthesis are steadily decreasing, this is likely the method of choice in future.

RESPONSE: We have expanded the text to include the mention of synthesis in pieces followed by assembly using methods such as Golden Gate. We have also added an example of when this approach is still inefficient in comparison to recombineering (lines 78-85). As a lab we have tried to have multiple genes synthesized and found that in several cases the GC content, size and complexity make it very challenging, timely and costly. Our developed recombineering pipeline circumnavigates this and is rapid.

Point 2. Page 6, top: how sure is it that the very large genes in Chlamydomonas are correctly annotated? Perhaps this can be addressed by comparison with genes in Volvox and Gonium?

RESPONSE: We agree that gene models in Chlamydomonas potentially could be incorrect, especially for larger genes. Comparison of average gene size across closely related Green algae shows relatively large variation (Chlamydomonas: 5322 bp, *Gonium pectorale*: 3990 bp and *Volvox carteri*: 6265 bp). However, to address the point raised by the reviewer a detailed gene vs. gene analysis would be required that we think is outside the scope of the manuscript. We would like to add that in many cases, recombineering would still allow correct cloning and localization of gene products in comparison to gene synthesis, where correct gene models (particularly correct translation initiation ATG, and intron/exon boundaries) would be critical for correct design of synthesis target.

These points are highlighted in lines 219-220 and 257-258. In addition, through recombineering we provide evidence that the gene model for Cre14.g613950 (described above in the response to the Editors notes) is potentially incorrect and that gene synthesis based on the current gene model would potentially have resulted in erroneous data (lines 490-494).

Point 3. I am missing a confirmation that the localization of the five example genes in Figure 4 is not the result of the in-frame integration of a broken construct into another gene. This could be addressed by providing information on how many independent transformants gave the same localization results, RT-PCR, or proteomics on a YFP pull down.

RESPONSE: All localisations were validated with two independently selected lines; we have provided images of a second independent transformant for each line in Figure S2 and added details of this in the Methods (lines 696-699). We also address this by performing immunoblotting against the 3xFLAG tag at the C-terminal of fusion proteins (Figure S2).

Point 4. Figure 5D: How many independent transformants have been tested here? This information is important, as the expression could be driven by another promoter, if the construct got disrupted and integrated into another gene (promoter trap). In this line, the authors show expression results only for a single gene, LCIB. Is this enough to extrapolate to all other promoters?

RESPONSE: We have now tested this for two additional independent LCIB transformants and see the same CO_2 response across all three lines. We have added additional bar charts to Figure 5E (formerly Figure 5D) and amended the text slightly to take into account the new data (lines 537-540).

Point 5. The authors mentioned that "57 of the 298 successfully cloned genes from Mackinder et al. (2017) contained a class 1 in-frame ATG upstream of the cloned region, therefore ~10% of cloned regions may have encoded truncated protein products". It would have been interesting to revisit these cases with genes cloned with the pipeline presented here to verify correct localization.

RESPONSE: We have amended the Results section to include mention of the overlap of 2017 and 2020 targets (lines 374 to 380). We also remark that of the successfully cloned targets in 2017 that contain a predicted class 1 uORF, five were retried in 2020 and all were successfully cloned (lines 393 to 394). Our method thereby provides the research community with a route for confirming localization of gene products whose sequences have an unclear transcription start site according to the genome annotation.

<u>Reviewer #2:</u>

Point 1. A major concern for the development of synthetic biology tools is to standardize the tools to enable modularity. Despite the clear interest of the recombineering pipeline presented, I regret that it lacks modularity. It would have been interesting to develop tools compatible with other standardized tools developed for Chlamydomonas, such as the Chlamydomonas Moclo Toolkit (Crozet et al. 2018, ACS Synth Biol). The nuclear sequences obtained through recombination with BAC would not be standardized (i.e. not domesticated due to the presence of undesired restriction sites) but standardization could have been used to design the recombineering vector tools in order to enable compatibility with all the MoClo bricks previously developed. This would broaden the possibilities of the pipeline by enabling the use of alternative bricks including alternative promoters, antibiotic resistances or tags.

RESPONSE: We recognize the importance of modularity in synthetic biology and as a lab are active users of Golden Gate cloning and the Chlamydomonas MoClo toolkit. During development of our recombineering pipeline, we considered MoClo compatibility but, as highlighted by the reviewer, analysis of the genome shows that the majority of genes would have to be domesticated (i.e. contain BsaI and BpiI restriction sites). I agree compatibility to backbone vectors could be of some use to the community but most vector parts (i.e. markers, fluorescence tags etc.) are available as part of the MoClo kit. One area we did overlook was the potential to assemble various backbone vectors using MoClo parts which could then be used in our recombineering pipeline. This is something we are considering to address in future work and would require the development of parts containing the ccdB counterselection gene and the unique I-SceI restriction site.

Point 2. The beginning of the Results section underscores problems in cloning Chlamydomonas genes but assumes that the only way to do so is through a PCR starting from the annotated ATG and ending at the annotated STOP. One can perfectly clone the entire genomic region through assembly of multiple parts generated by PCR or gene synthesis. For example, Golden Gate cloning can be a powerful tool to assemble scarlessly in one step multiple fragments obtained by PCR or synthesis. These points could be discussed in the manuscript.

RESPONSE: A similar point was raised by Reviewer #1. We agree with both reviewers and have now addressed this in the Introduction (lines 78-79).

Point 3. The genomic analyses provided in the Results section are quite interesting but lack precision on how the data were generated or where they come from. For instance, are the position of introns based on gene models or were some introns/gene models modified (e.g. annotated from cDNA/RNAseq analysis)? This should be more clearly stated.

RESPONSE: We used gene annotation data from the Chlamydomonas genome annotation version 5.5. Underlying gene structure is predicted using Augustus and a diverse range of RNAseq datasets are used to refine gene predictions. We have now clarified this in the Methods section (lines 801-824).

Point 4. The authors should clarify the usage of some words. For example, the term "gene" refers to 1/ the genomic intron-containing sequence encompassing the promoter, the coding sequence and UTRs (section in 'recombineering pipeline development') or 2/ only the transcribed regions (for instance in fig1F) or 3/ only the coding sequence (section 'gene complexity'). This should be corrected.

RESPONSE: We have amended the 'Analysis of the Chlamydomonas genome highlights the challenges affecting PCR-based cloning' Results section to clarify this (lines 157, 176, 193, 216). The whole genome analysis of repeat frequency was conducted on nuclear genes measured from the start of the 5'UTR to the end of the 3'UTR (lines 191-193). We have also added a note in the 'genome analysis' Methods section to clarify this (lines 790-792). We have amended the legend for Figure 1F to clarify that our comparison of gene size and repeat frequency between species measures genes from the start of the 5'UTR to the end of the 3'UTR.

We have modified the 'Recombineering pipeline development' Results section to clarify that our pipeline clones the native promoter region plus 5'UTR plus open reading frame (lines 314-315), which is further explained in the Discussion (lines 605-606).

<u>Reviewer #3:</u>

Point 1. It might be better to condense the Introduction; it is a little repetitious and sometimes contains more detail about the CCM and the methods than is necessary. This applies to the abstract and Results to some extent as well... for example, is the sentence 'We initially applied the pipeline to 12 targets with a 92% cloning success rate' necessary in the abstract. In the Results under 'Genetic complexity' is the first sentence necessary? There are additional places that would benefit from some pruning.

RESPONSE: We have pruned the abstract by combining the batch and high-throughput data (lines 37-39). We have removed unnecessary detail from the Introduction about the CCM (lines 128-132). We have shortened the first sentence in the 'Gene complexity" section (line 188-189).

Point 2. The Introduction discusses the 2017 Mackinder papers in which an attempt was made to clone 624 gDNAs encoding proteins with putative CCM functions (successfully cloning 298). The relationship between the 191 genes used to test the recombineering method and the original 624 genes is not absolutely clear. Did the current test population only contain genes that were not successfully cloned by the PCR based approach (we are told it is 'primarily' from the CCM gene population examined in 2017)? Is there any particular reason why these genes were chosen (rather than using all 326 genes not successfully cloned in the earlier work; or were 135 of the 326 genes not represented in the BAC library)?

RESPONSE: We have modified the Results section to clarify the relationship between our 2017 targets and the 191 recombineering targets; only 81 targets from 2017 were included in our recombineering shortlist, with the remainder being selected based on newer pyrenoid proteome and CCM protein-protein interactome data (lines 374 to 380).

Point 3. I am not sure that the sentence 'For the 146 correctly recombineered lines, picking just a single colony gave a 63% success rate, screening a second colony increased the success rate to 85% and a third colony gave a 97% success rate, for a small proportion of targets screening >3 colonies led to the identification of a correctly recombined construct (Figure 2E)' is structured properly. I am assuming that the 14 cases in which a different BAC (harboring the same target gene) had to be chosen to make the procedure work were part of the 146 that worked (so initially 132 worked with 14 additional successes when another BAC was used?).

RESPONSE: We have modified this sentence to clarify the success rates based on picking one, two, three or more colonies (lines 398-403).

Point 4. The numbers are a little confusing; in the Introduction it says that the success in cloning the genes was 77%, which is ~146 genes, which is what it indicates in the results. However, toward the end of the Discussion it says 157 CCM genes were successfully cloned.

RESPONSE: This confusion comes from us previously presenting the small scale (11 out of 12 targets) and the large-scale (146 out of 191 targets) results separately. In the abstract and Introduction we now refer to the combined results (157 out of 203 targets). We have also added a sentence to the Results clarifying that out of all targets attempted (in both small and large scale attempts) the overall cloning efficiency is 157/203 = 77% (lines 418-419).

Point 5. The BAC library is from CC-503 while the strain used for transformation is CC-4533. Are there any difficulties that should be considered when using these different resources? For example, is there information indicating that the two strains show identical acclimation responses (same CCM genes upregulated?).

RESPONSE: To address this, we have added a subsection to the "Genome analysis" section in the Methods titled: *"Note on differences between Chlamydomonas BAC library strain and CLiP mutant strain"* that highlights genetic differences (lines 925-944).

To our knowledge, there is no direct transcriptomic comparison of CCM induction between CC-503 and CC-4533. Both strains have been widely used to study the CCM.

Point 6. Also, unless I missed it on the website, it would be worth providing the Resource Center with a protocol for the recombineering (emphasizing critical aspects, including the growth of the bacteria for transformation with the recombineering cassette), availability to the plasmids used and access to important software, such as BACSearcher (everything to make it easy for most labs to effectively use the technique).

RESPONSE: We have now deposited all plasmids to the Chlamydomonas Resource Center. We also clearly outline the availability of all the materials, data and software in a dedicated Methods section (lines 651-659).

Point 7. It might be worth providing a little more information about the repeats, which I am sure can be readily determined. What is the distribution of mono, di, tri, poly, tandem, inverted repeats in the different domains of the genes (coding region, intron, exon, UTRs)? Are the inverted repeats, which appear in most genes, primarily in the 3' ends? Is there any correlation between the position in the gene and type of repeat? Is there a stronger correlation between cloneability with the type/position of the repeats than just with the presence of repeats? This might provide more information about both the structure of the genome and the factors that impact the ability to clone a gene.

RESPONSE: We have amended Figure 1B to report repeat prevalence in the 5'UTR, ATG-Stop and 3'UTR (also see lines 211-214). However, we feel that further detailed information about the distribution of repeats within genes, while interesting, is outside the scope of the study and doesn't contribute significantly to our reporting of the recombineering methodology.

Point 8. Some examples are given for validation of the localization of the fluorescence tagged proteins, although it hasn't been done with the detail needed to absolutely confirm proper localization. It is likely that PSAF is properly localized (at least to the thylakoid membranes), but the introduced PSAF was not shown to rescue a mutant strain or shown to be associated with PSI particles; it could be in thylakoids and yet not be properly localized or functionally competent. The same types of arguments can be made for the TAB2 and Cre14.g613950 and Cre10.g435800. To discuss the validity of the localization it would be best to show that the fusion proteins rescue the mutant strains, locate to a particular compartment/complex in those strains, and then show that the localization is exactly the same in wild type cells. I am suggesting a little more caution in interpreting the localization data if there is really no check that

the protein is functioning properly in the transformants... although I do agree that given prior observations the conclusions are reasonable.

RESPONSE: We agree with the reviewer that without performing mutant complementation with the fusion protein, a degree of caution needs to be taken with the localization. Although, as acknowledged by the reviewer, the supporting data (i.e. proposed function, localization to the same subcellular domain as interactors and proteomic data) all strongly support the localization data.

Performing complementation analysis for all shown target genes would be challenging and potentially face several hurdles. It is unclear if mutants in the chosen genes would have clear associated phenotypes. It would also rely on mutant availability, mutant genotype confirmation and confirmation of the absence of translated protein. Although interesting, we feel that this is outside the scope of the work and would take a considerable time to perform for all examples. However, in line with the reviewer, we agree that it is important to validate that recombineered targets fused to a fluorophore can be used to rescue a CLiP mutant and that localization is unaffected. For this we demonstrate successful complementation of a CLiP mutant in *LCIB* and show a localization pattern of LCIB-Venus that is consistent with expression in WT (see Figure S3 and lines 518-523)

Point 9. In the section of the Results on 'Maintaining the native promoter....' the way it is written may be improved a bit. For example, the first sentence in the paragraph 'As our pipeline retains the native promoter of the target gene we hypothesized that fluorescence output would be representative of relative protein abundance' seems a little confusing. Even if the target gene didn't have the native promoter, you might hypothesize that the fluorescence output would represent the relative protein abundance. I guess the authors are basically saying that constructs with the native promoter and a fluorophore fusion could be used to monitor protein accumulation (e.g. potentially reflecting changes in gene expression) that occur when environmental conditions are changed, such as changes in expression of genes encoding CCM proteins.

RESPONSE: We agree with the reviewer that the previous wording was confusing. We have amended the text to clarify this (lines 534-535).

TPC2020-LSB-00363R1 2nd Editorial decision – *accept with minor revision*	Dec. 9, 2020

On the basis of the advice received, the board of reviewing editors would like to accept your manuscript for publication in The Plant Cell. This acceptance is contingent on revision based on the comments of our reviewers. In particular, we ask you to pay attention to the useful suggestions of Reviewer 1 to further improve the abstract and the text of your manuscript. The reviewer suggests highlighting the importance of the described recombineering pipeline not only to complement mutants of the CLiP collection, but also mutants generated by other methods (e.g., CRISPR/Cas technology). This aspect could also be emphasized in the Discussion.

RESPONSE: We agree with this change and have amended the abstract (see below) and have added the following to the Discussion, lines 549-550: "complementation of mutants (e.g. random insertion and CRISPR/Cas generated mutants)."

Reviewer #1 (Comments for the Author):

The authors have carefully and convincingly addressed the points I had raised. I very much like the extension of the method to fosmid libraries. I still have a few points I would want to ask the authors to address, which does not require an additional reviewing loop: - In the Abstract, the authors focus too much on the complementarity of their method with mutants generated in the Chlamydomonas Library Project. Of course, this is valid, but I find it too exclusive. More and more Chlamy labs have established the CRISPR/Cas technology to generate knock-out mutants. Since employed gRNAs may have off-target effects, complementation might be required here, as well, and the recombineering approach would be very well suited. So perhaps simply add an "e.g." ahead of "Chlamydomonas Library Project". Also include "other algal and plant species".
- Line 407: Please provide some numbers on the fraction of transformants expressing the construct. If e.g. a 3xHA epitope is used, screening cannot be done based on fluorescence and may require extensive screening efforts. Was CC4533 always used? Did you also use UVM4/11?

Reviewer #2 (Comments for the Author):

The authors have carefully addressed all the comments raised by the reviewers. The revised manuscript is significantly improved.

Reviewer #3 (Comments for the Author):

The authors have done an excellent job addressing the issues raised, have clarified the text in a number of places and have added additional information that makes the manuscript more valuable, including the use of fosmids and additional information concerning access to the resources.

**TPC2020-LSB-00363R2 2nd Revision received**	**Dec. 18, 2020**

Reviewer comments and **author responses**:

Reviewer #1:

Point 1. In the Abstract, the authors focus too much on the complementarity of their method with mutants generated in the Chlamydomonas Library Project. Of course, this is valid, but I find it too exclusive. More and more Chlamy labs have established the CRISPR/Cas technology to generate knock-out mutants. Since employed gRNAs may have off-target effects, complementation might be required here, as well, and the recombineering approach would be very well suited. So perhaps simply add an "e.g." ahead of "Chlamydomonas Library Project". Also include "other algal and plant species".

> **RESPONSE: We thank the Reviewer for highlighting this and fully agree with the point raised. We have modified the abstract as suggested, lines 41-43: "To expand the functionality of our system, we developed a series of localization vectors that enable complementation of mutants (e.g. Chlamydomonas Library Project and CRISPR/Cas generated mutants) and enable protein tagging with a range of fluorophores."**
>
> **We have added "and plant" to line 45.**

Point 2. Line 407: Please provide some numbers on the fraction of transformants expressing the construct. If e.g. a 3xHA epitope is used, screening cannot be done based on fluorescence and may require extensive screening efforts. Was CC4533 always used? Did you also use UVM4/11?

> **RESPONSE: We have now added this information. We decided to add it to the Methods section so as not to break the flow of the Results. Lines 599-602: "The average number of fluorescent colonies for recombineered Venus fusion proteins with their native promoter was ~10%, however this varied considerably between constructs (PSAF (10/134) 7%, TAB2 (6/44) 13.6%, CSP41B (6/43) 13.9%, ISA1 (25/297) 8%, Cre14.g613950 (2/22) 9%, LCI9 (6/25) 24%, LCIB (6/19) 31.5%)." CC4533 was always used, we did not use UVM4/11.**

**TPC2020-LSB-00363R2 3rd Editorial decision – *acceptance pending***	**Dec. 18, 2020**

We are pleased to inform you that your paper entitled "A recombineering pipeline to clone large and complex genes in Chlamydomonas" has been accepted for publication in The Plant Cell, pending a final minor editorial review by journal staff. At this stage, your manuscript will be evaluated by a Science Editor with respect to its presentation of scientific content, compliance with journal policies, and presentation for a broad readership.

**Final acceptance from Science Editor**	**Jan. 18, 2021**
